# PDS5A restricts SYN4-dependent long-range chromatin contacts to shape TAD-like chromatin architecture in *Arabidopsis*

**DOI:** 10.1093/plcell/koag325

**Published:** 2026-10-27

**Authors:** Sofia Tzourtzou, Zhidan Wang, Sarah Leuchtenberg, Priyanka Kumari, David Latrasse, Moussa Benhamed, Chang Liu

**Affiliations:** Department of Epigenetics, Institute of Biology, University of Hohenheim, Garbenstrasse 30, Stuttgart 70599, Germany; Department of Epigenetics, Institute of Biology, University of Hohenheim, Garbenstrasse 30, Stuttgart 70599, Germany; Department of Epigenetics, Institute of Biology, University of Hohenheim, Garbenstrasse 30, Stuttgart 70599, Germany; Department of Epigenetics, Institute of Biology, University of Hohenheim, Garbenstrasse 30, Stuttgart 70599, Germany; CNRS, INRAE, Univ Evry, Institute of Plant Sciences Paris-Saclay (IPS2), Université Paris-Saclay, Orsay 91405, France; CNRS, INRAE, Univ Evry, Institute of Plant Sciences Paris-Saclay (IPS2), Université Paris-Saclay, Orsay 91405, France; Institute of Plant Sciences Paris-Saclay (IPS2), Université de Paris Cité, Gif-sur-Yvette 91190, France; Department of Epigenetics, Institute of Biology, University of Hohenheim, Garbenstrasse 30, Stuttgart 70599, Germany; Cluster of Excellence GreenRobust, University of Hohenheim, Stuttgart 70599, Germany

## Abstract

Three-dimensional genome folding in vertebrates, such as the formation of topologically associating domains (TADs), depends on cohesin-mediated loop extrusion, yet its regulation in plants remains unresolved. Using Hi–C and Micro-C, we show that *Arabidopsis* cohesin regulator PDS5A is a key modulator of chromatin loop extrusion and TAD-like architecture. The TAD-like domain patterns in *pds5a* are highly similar across tissues and ploidy levels. Genetic evidence reveals that TAD-like domains require the plant kleisin SYN4 and are opposed by WAPL1/2, with PDS5A serving as the major cohesin-unloading factor. Biochemical assays show that PDS5A associates with cohesin independently of its Tudor domain-mediated H3K4me1 binding. Micro-C further identifies accessible, highly transcribed promoters as chromatin loop and stripe anchors. Finally, mutational disruption of distal chromatin anchors shows that newly formed chromatin contacts in *pds5a* can act as cis-regulatory modules that influence target gene expression. Together, these findings identify a PDS5A-regulated chromatin extrusion module that contributes to plant 3D genome organization and uncover a promoter-based logic for loop anchoring and transcriptional control in plants.

## Introduction

The current understanding of eukaryotic genome organization highlights the profound impact of three-dimensional (3D) chromatin architecture on gene expression, DNA replication, and genome integrity (Rowley and Corces 2018; Hafner and Boettiger 2023; Giles et al. 2025). In vertebrates, topologically associating domains (TADs) represent a structural organizational unit of the genome, which are demarcated by increased frequencies of internal chromatin interactions and bounded by regions of strong insulation (Dixon et al. 2012; Nora et al. 2012; Rao et al. 2014). Recent advances have revealed that plant genomes, though lacking canonical vertebrate insulator proteins, also exhibit spatial compartmentalization at sub-chromosomal levels, including the presence of so-called “TAD-like” domains (reviewed by Domb et al. 2022; Tourdot et al. 2023; Lee and Seo 2025; Xiao et al. 2025). However, mechanisms underlying their establishment and regulation in plants remain poorly understood.

TADs in mammals are contiguous genomic regions featuring high levels of intra-domain contacts and sharp interaction decreases at domain boundaries, visible as triangles along the diagonal in Hi–C contact maps (Dixon et al. 2012). TAD boundaries are often enriched for transcription start sites (TSSs), housekeeping genes, active promoters, and specific architectural proteins such as CCCTC-binding factor (CTCF) and cohesin complexes (Dixon et al. 2012; Rao et al. 2014; Szabo et al. 2019). Biochemical and genetic studies in animals have established that some TADs in the genome are essential for regulatory insulation, with their perturbation leading to aberrant gene expression and disease phenotypes (Lupianez et al. 2015; Flavahan et al. 2016; Valton and Dekker 2016; Rajderkar et al. 2023).

The formation of vertebrate TADs has been traditionally explained by the loop extrusion model, wherein the ring-shaped cohesin complex loads onto DNA and, powered by ATP hydrolysis, translocates along chromatin to extrude loops of increasing size (Rao et al. 2014; Sanborn et al. 2015; Vian et al. 2018; Guerin et al. 2024; Rittenhouse and Dowen 2024). This process continues until cohesin encounters convergently oriented CTCF binding sites, which serve as a barrier, thereby fixing the boundaries of TADs. The importance of cohesin and CTCF is underscored by genetic depletion experiments: loss of either protein disrupts TAD structure, despite leaving higher-level compartmentalization relatively intact (Haarhuis et al. 2017; Rao et al. 2017; Schwarzer et al. 2017; Liu et al. 2025). Additional contributors to domain formation in mammals include transcriptional activity and chromatin state, as some boundaries persist even in the absence of CTCF or cohesin (Ulianov et al. 2016; Rowley et al. 2017; Banigan et al. 2023; Szalay et al. 2024). In yeast, which lacks CTCF, cohesin-mediated loop extrusion can be terminated by collision with other cohesin complexes rather than sequence-specific barriers, highlighting potential alternative mechanisms conserved among eukaryotes. In this scenario, cohesin-associated regions, which are often located at TTSs of convergently transcribed genes, act as stalling points for extrusion (Higashi et al. 2021). Notably, loop extrusion mechanisms also create features other than TADs. One of them is called “architectural stripes”, which are line-like interaction patterns detected in Hi–C maps (Vian et al. 2018). They are thought to reflect the persistent interaction of an extrusion complex with a chromatin anchor, producing a characteristic streak extending from the anchor site. Stripes are often linked to active chromatin and regulatory elements, and their occurrence can provide clues about local genome organization and potential regulatory interactions (Vian et al. 2018; Yoon et al. 2022; Chen et al. 2023).

Plant genomes display complex and diverse 3D chromatin organizations. Many plants exhibit chromatin interaction patterns on Hi–C maps that are similar to those associated with mammalian TADs, in this work, we use “TAD-like domains” to refer to them in general. TAD-like domains, with sizes of tens to hundreds of kb, have been found on Hi–C maps of crops with large genomes (eg, rice, tomato, maize, wheat and cotton), as well as in small genome species like *Marchantia polymorpha* (Dong et al. 2017; Liu et al. 2017; Concia et al. 2020; Karaaslan et al. 2020; Wang et al. 2021). Plant TAD-like domain boundaries are often enriched in transcriptionally active regions and active histone marks; however, typical long-range border-to-border contacts, which are hallmarks of mammalian TADs, are less pronounced. In the dicot model plant *Arabidopsis thaliana,* which has a smaller genome compared to other plants, TAD-like domains are unrecognizable. However, *Arabidopsis* domain-like structures, containing 1 or up to a few genes, are visible on high-resolution contact maps normalized at 1 kb or sub-kb windows (Lee and Seo 2023; Yin et al. 2023; Sun et al. 2024). These observations have fostered debate about whether plant TAD-like domains arise from the same regulatory mechanisms as mammalian TADs (Dogan and Liu 2018; Stam et al. 2019).

In plants, clear CTCF homologs are absent, yet core cohesin components and candidate regulators are conserved (Szalay et al. 2024). Several plant-specific DNA- and protein-based barriers have been proposed to modulate domain boundaries, such as regulators of Polycomb group proteins (eg, PWWP-DOMAIN INTERACTOR OF POLYCOMBS1 (PWO1) and EMBRYONIC FLOWER 1 (EMF1)) (Yin et al. 2023; Shu et al. 2024; Yang et al. 2024). However, disruption of these proteins has not led to the complete loss of TAD-like demarcation, suggesting further mechanistic complexity. Superimposed on these potential boundary factors, changes in epigenomic modifications can modulate local domain formation in plants but are often insufficient to fully explain TAD-like architecture (Huang et al. 2021; Wang et al. 2022; An et al. 2024; He et al. 2024; Shu et al. 2024).

Recently, we found that mutation of PDS5A, which encodes a coregulator of cohesin, remarkably enhanced TAD-like structures in the *Arabidopsis* genome (Göbel et al. 2024). This finding raised fundamental questions about the evolution of chromosome architecture in eukaryotes and the extent to which transcriptional activity, epigenomic states, and mechanical constraints jointly shaped domain organization in plant nuclei. Here, we present a systematic identification of cohesin subunits and regulatory factors that govern TAD-like domain formation in *A. thaliana*. Furthermore, we identify and characterize *Arabidopsis* chromatin anchor regions that serve as loop anchors and mediate long-range cis-regulatory communication.

## Results

### PDS5A-dependent TAD-like domain architecture in *Arabidopsis* is conserved across tissues and ploidy levels

In our recent study, we demonstrated that PDS5A functions as a key regulator of chromatin organization in *Arabidopsis* by suppressing the formation of TAD-like domains at the genomic level. To further elucidate the mechanisms underlying TAD boundary establishment in the *pds5a* mutant, we investigated several potential contributing factors, such as transcriptional reprogramming, endopolyploidy levels, and genetic interactions.

We began by comparing Hi–C maps generated from leaf, root, and germinating seed tissues, which represent distinct developmental stages and gene expression profiles. In total, 1,185 TAD-like domains were identified from *pds5a* leaf tissues, which were hardly recognizable in the corresponding Hi–C maps of wild-type samples (Fig. 1a, Data Set S1 and S2). Notably, *pds5a* TAD-like domains are substantially larger than the compartment domains (CDs) recently identified in wild-type plants and span a greater proportion of the genome, suggesting that these 2 types of domains are formed through distinct regulatory mechanisms (Yin et al. 2023; Shu et al. 2024) (Figure S1). The enhancement of TAD-like domains in *pds5a* is accompanied by the strengthening of local chromatin insulation (Göbel et al. 2024). Insulation scores were calculated to quantify potential changes in chromatin insulation across samples (Crane et al. 2015), which indicated that chromatin insulation in leaves and roots was highly similar, whereas it was weaker in germinating seeds (Fig. 1b and c). Aggregate TAD plots, which depict normalized observed/expected chromatin contacts within and across TAD-like domains, revealed that these structures in *pds5a* leaf and root tissues were insulated similarly from their neighboring regions, but such insulation was weakened in germinating seeds (Fig. 1d). Such differences in chromatin insulation in germinating seeds may reflect the pronounced reorganization of nuclear architecture that occurs during germination, including nuclear expansion and heterochromatin reorganization (Bourbousse et al. 2015). We also examined the patterns of TAD-like domains in leaves of heat-stressed plants, as heat stress is known to induce changes in chromatin organization at a chromosomal level, for instance, dissociation of KNOT ENGAGED ELEMENT (KEE) regions that form megabase-sized chromatin loops (Grob et al. 2014; Sun et al. 2020). In heat-stressed *pds5a* leaves, we observed complete dissociation of KEEs (Figure S2), but TAD-like domains at the local level appeared unchanged in comparison to untreated leaves, suggesting that chromatin interactions at KEE loci in *Arabidopsis* are independent of *PDS5A* (Fig. 1e).

**Figure 1:**
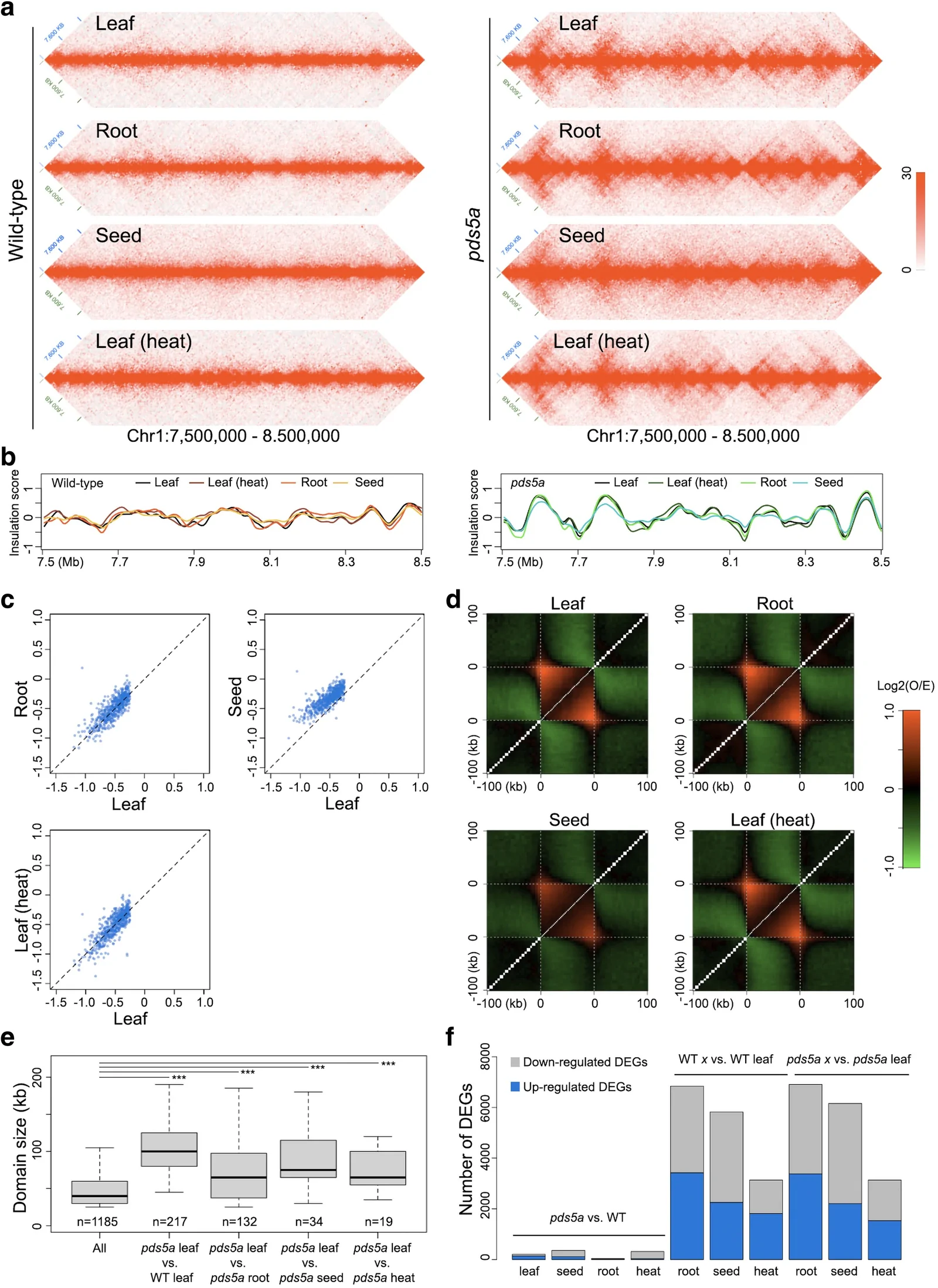
Comparative analysis of chromatin insulation, TAD-like domains, and gene expression in *pds5a*. a) and b) Comparisons of wild-type and *pds5a* Hi–C contact maps (a) and the corresponding profiles (b), both calculated at 5 kb resolution for a 1 Mb region of chromosome 1. Regions with local minima in the insulation score indicate positions of strong chromatin insulation. c) Correlation of genome-wide insulation scores between different *pds5a* samples. Scatter plots display insulation scores specifically from regions exhibiting strong chromatin insulation. d) Metagene plot depicting relative chromatin contact strengths within *pds5a* TAD-like domains and their 100 kb flanking regions. Relative chromatin contact was calculated as the log2 ratio of observed versus expected contact frequencies (log2(O/E)), based on normalized Hi–C matrices. e) Size distribution of reorganized domains. *** indicate statistical significance (*P* < 1 × 10^−4^) as determined by two-sided Wilcoxon rank-sum tests. f) Numbers of differentially expressed genes (DEGs).

We next applied differential domain analysis to assess changes in *pds5a* TAD-like domains across tissues. We defined a TAD-like domain as “reorganized” when its intra-domain interactions differed significantly between samples (see Methods). Among the 1,185 TAD-like domains identified in *pds5a*, 217 were classified as reorganized in wild-type leaves. These reorganized domains tended to have larger sizes, consistent with the observation that the *pds5a* mutation promotes long-distance chromatin interactions. Across *pds5a* samples, we observed varying degrees of TAD-like domain reorganization, with the fewest changes detected in heat-stressed leaves (19 differential domains). Overall, in *pds5a* samples, more than 1,000 domains remained unchanged, indicating that most TAD-like domains are largely stable (Fig. 1e, Data Set S2).

Next, we profiled gene expression in these samples and examined its relationship to TAD-like domains. Notably, far fewer differentially expressed genes (DEGs) were associated with the *pds5a* mutation than with differences in tissue identity or growth conditions (Fig. 1f, Data Set S3), suggesting that TAD-like domains have a relatively limited impact on the transcriptome. Given the substantial transcriptomic differences among distinct *pds5a* samples compared with *pds5a* leaves, we further investigated whether DEGs are preferentially localized within, outside, or at the boundaries of TAD-like domains. This analysis showed that only a small fraction of DEGs reside in differential TAD-like domains, and DEGs do not display a significant positional bias with respect to TAD-like domains (Figure S3a and b). Likewise, chromatin regions containing DEGs did not show significant alteration in their insulation scores (Figure S3c–e).

Endoreduplication, a process in which cells replicate their genomic DNA without undergoing subsequent cell division, is a common phenomenon in *Arabidopsis* (Sugimoto-Shirasu and Roberts 2003; Tourdot et al. 2023). In 2-wk-old *Arabidopsis* seedlings that grow under standard conditions, approximately half of the nuclei had undergone at least 1 endocycle (Figure S4a). To clarify whether *pds5a* “TAD-like domain” patterns were attributed to sister chromatid contacts in endoreduplicated nuclei, we generated endopolyploidy-specific Hi–C maps (Hu et al. 2019). Our results demonstrated that 2C nuclei (ie, without endoreduplication) had similar TAD-like domain patterns compared to 4C and 8C nuclei, excluding the participation of endoreduplication-specific mechanisms underlying TAD-like domain formation in *pds5a* (Figure S4b). Taken together, our results indicate that TAD-like domains in *Arabidopsis* show highly similar patterns across different tissues and are largely independent of changes in gene expression.

### WAPL1/2 and SYN4 participate in regulating TAD-like domains

Constituent members of cohesin and its co-regulators that modulate cohesin-chromatin interaction dynamics are highly conserved in eukaryotes (Perea-Resa et al. 2021). Via extruding chromatin, the cohesin protein complex plays a pivotal role in defining vertebrate TAD structures (da Costa-Nunes and Noordermeer 2023). In plants, WAPL acts as a cohesin release factor that promotes cohesin dissociation from chromatin, thereby regulating cohesin dynamics and preventing excessive chromatin loop extension, while SYN2 and SYN4, which are orthologs of α-kleisin, act as cohesin subunits in somatic cells, known for maintaining proper cohesin function (Yuan et al. 2012; De et al. 2014). To examine their genetic interactions with *pds5a*, we generated higher-order mutant combinations and analyzed their chromatin architecture by Hi–C.

Compared with *pds5a* alone, *pds5a wapl1 wapl2* and *pds5a syn4* mutants displayed strikingly distinct intrachromosomal contact patterns. Loss of WAPL activity in the *pds5a* background caused a strong reinforcement of long-range chromatin interactions that extended across TAD-like domain boundaries, consistent with unrestrained loop extrusion (Fig. 2a). Notably, depletion of WAPL proteins in vertebrates produces comparable alterations in chromatin contact profiles (Haarhuis et al. 2017; Liu et al. 2025), underscoring a conserved role for WAPL in constraining cohesin activity (Figure S5). *pds5a syn4* mutants completely lacked detectable TAD-like domains, indicating that *SYN4* is indispensable for the formation of these chromatin structures (Fig. 2a). On the other hand, *syn2* mutation did not alter TAD-like domains in *pds5a* (Fig. 2a). Altogether, our results indicate that cohesin-mediated chromatin extrusion is dependent on *SYN4* but is suppressed by *PDS5A* and *WAPLs.*

**Figure 2:**
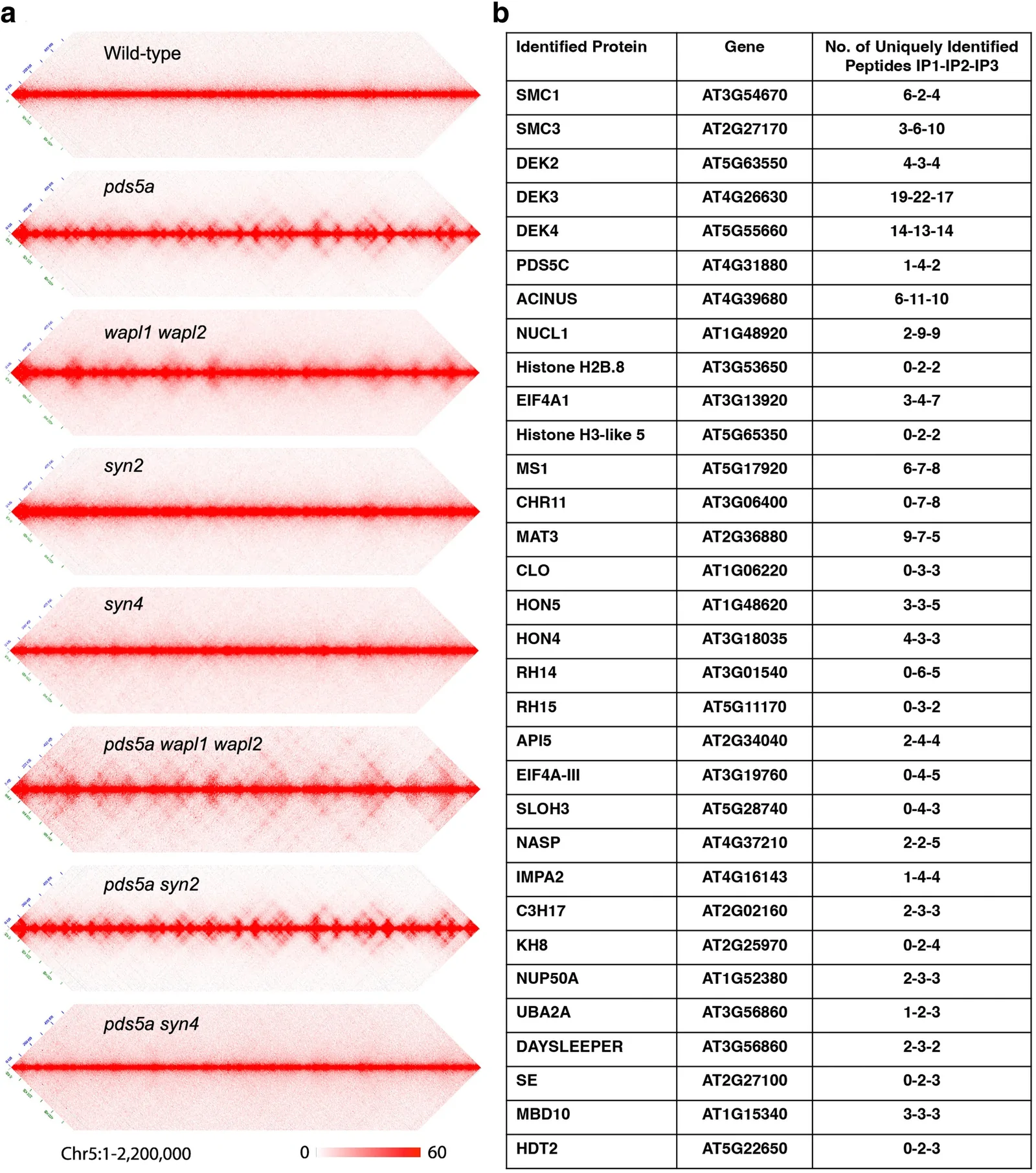
Involvement of cohesin in *Arabidopsis* chromatin loop extrusion. a) Comparison of Hi–C maps of different genotypes. The graphs illustrate Hi–C maps of a representative genomic region (2.2 Mb from chromosome 5), normalized at 5 kb resolution. The Hi–C map of *wapl1 wapl2* is from our previous work (Göbel et al. 2024). b) *In vivo* identification of PDS5A-associated proteins. PDS5A:2HA was immunoprecipitated from 14-d-old *Arabidopsis* shoot tissue using anti-HA magnetic beads, and co-purifying proteins were identified by mass spectrometry. Wild-type (WT) plants were used as controls. The table shows proteins identified in at least 2 replicates but not in WT.

### PDS5A interacts with cohesin

In non-plant species, PDS5 interacts with cohesin to promote cohesin release from chromatin as well as to prevent cohesin from establishing TAD boundaries (Chan et al. 2013; van Ruiten et al. 2022). To examine whether PDS5A associates with cohesin in *Arabidopsis* cells, we performed anti-HA immunoprecipitation coupled with mass spectrometry (IP-MS) using the *PDS5A:2HA* complementation plants. Wild-type tissue was processed in parallel as a negative control. This in vivo affinity purification analysis recovered the core cohesin subunits SMC1 and SMC3, and SYN4 was also detected among PDS5A-associated proteins (Fig. 2b; Data Set S4), supporting an association of PDS5A with cohesin complexes in *Arabidopsis* cells. To explore candidate pairwise contacts within this complex, we next performed yeast two-hybrid assays (Y2H). The N-terminal region of PDS5A interacted with SCC3 and with the tested C-terminal region of SMC3 (Fig. 3). The Y2H pattern is consistent with the possibility that PDS5A contacts cohesin through a region involving SMC3, reminiscent of PDS5-SMC3 associations described in non-plant systems (Hons et al. 2016). Together, these data support a model in which PDS5A associates with cohesin to constrain cohesin-dependent chromatin contacts in *Arabidopsis*.

**Figure 3:**
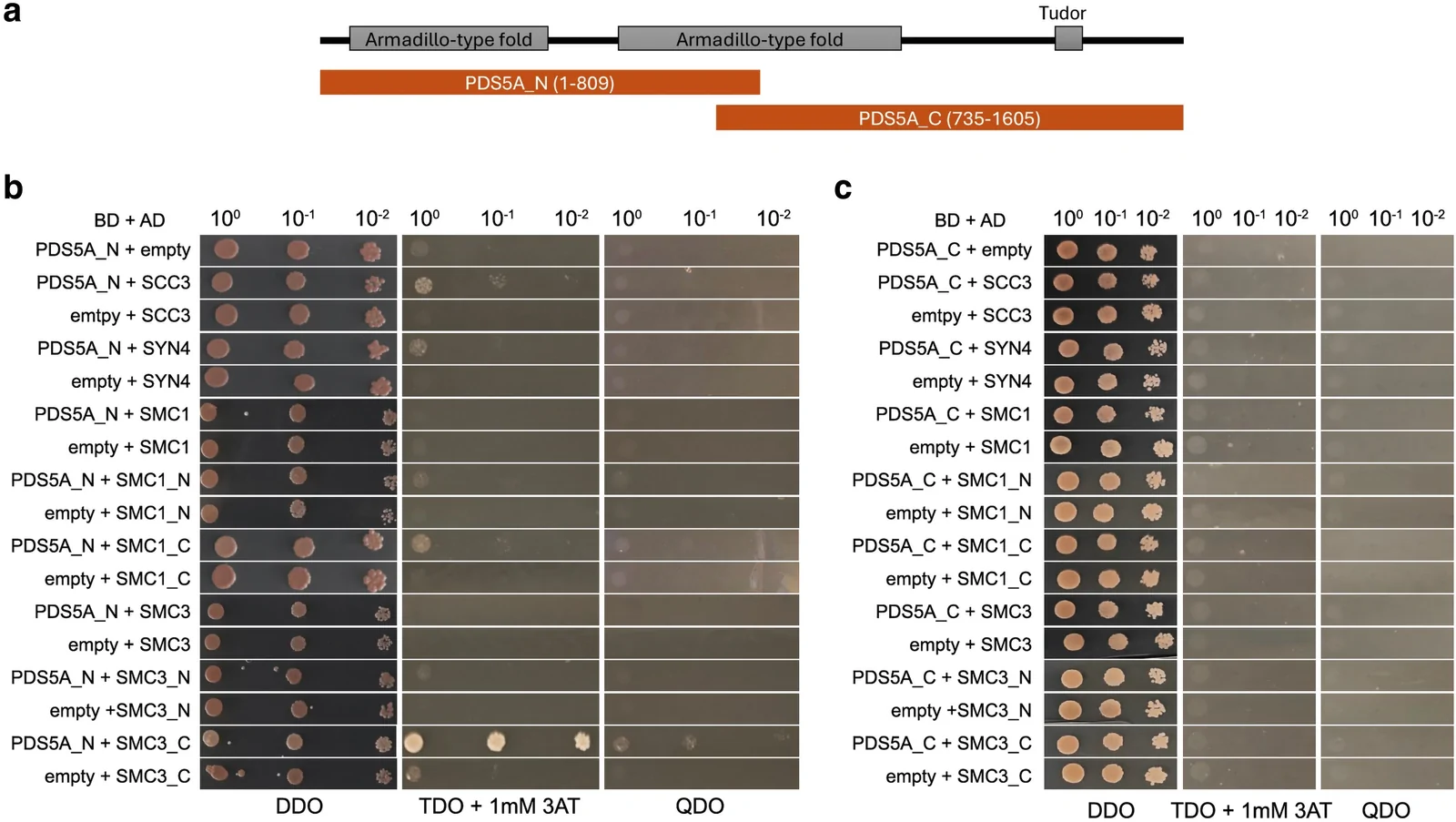
Yeast two-hybrid analysis of PDS5A interactions with cohesin complex proteins. a) Schematic representation of PDS5A constructs used in this study. Full-length PDS5A was divided into N-terminus and C-terminus fragments, which were fused to the GAL4 DNA-binding domain (BD) to generate bait constructs for interaction detection. b) Interaction analysis between the PDS5A N-terminal domain (PDS5A_N) and cohesin complex proteins SYN4, SCC3, SMC1, and SMC3 fused to the GAL4 activation domain (AD) in the Y2HGold strain. For SMC1 and SMC3, truncated N-terminal and C-terminal constructs were also tested. Co-transformants were selected on double dropout medium (DDO, SD -Leu/-Trp) and interaction-dependent growth was assessed on more stringent triple and quadruple dropout medium (TDO+1 mM 3-AT, SD -Leu/-Trp/-His; QDO, SD -Leu/-Trp/-His/-Ade). Serial dilution spot assays (10^0^ to 10^−2^) were performed to compare the relative strengths of interactions. Empty vector controls were included to monitor background growth and autoactivation. c) Interaction analysis as in (B), using the PDS5A C-terminal domain (PDS5A_C) against the same set of cohesin proteins under identical selective conditions with empty vector controls.

### Tudor domain-mediated chromatin binding of PDS5A is dispensable for chromatin folding regulation

Based on sequence homology, PDS5A was proposed to regulate genome organization through chromatin binding mediated by its Tudor domain, as the Tudor domain of PDS5C was shown to recognize histone H3K4 monomethylation (H3K4me1) (Niu et al. 2021; Göbel et al. 2024). In light of this, we performed chromatin immunoprecipitation sequencing (ChIP-seq) experiments to characterize potential PDS5A-chromatin interactions (Figure S6). We found that the majority of PDS5A-bound chromatin regions (83%) overlapped with those marked with H3K4me1, and these PDS5A-chromatin interactions showed a positive correlation with the degree of H3K4me1 marks (Fig. 4a and b, Figure S6b and c). To verify whether the Tudor domain is required by PDS5A to interact with chromatin, we created a construct containing a *PDS5A:2HA* variant (*PDS5A:2HA^3A^*) carrying 3 point mutations in the Tudor domain predicted to disrupt H3K4me1 binding (Niu et al. 2021; Quiroz et al. 2024) and expressed it in the *pds5a/b* background at levels comparable to the complementation line (Fig. 4c). Clearly, ChIP-seq experiments indicated that the mutated PDS5A:2HA lost most of the binding sites and could not bind to H3K4me1-marked chromatin (Fig. 4a and b, Figure S6d). Furthermore, in comparison to PDS5A:2HA, the interactions between the mutant variant and chromatin were significantly weaker (Figure S6e), indicating that the Tudor domain is essential for PDS5A-chromatin interactions in vivo. Surprisingly, Hi–C analysis revealed that PDS5A:2HA^3A^ fully restored TAD-like domain patterns in *pds5a/b* to wild-type levels (Fig. 4d and e). Interestingly, our inspection of PDS5A-chromatin interactions around TAD-like borders, which show enhanced chromatin insulation in *pds5a* mutants, did not reveal specific patterns (Figure S6f and g). Thus, PDS5A's role in constraining TAD-like domain formation appears to be independent of direct Tudor domain-mediated chromatin binding.

**Figure 4:**
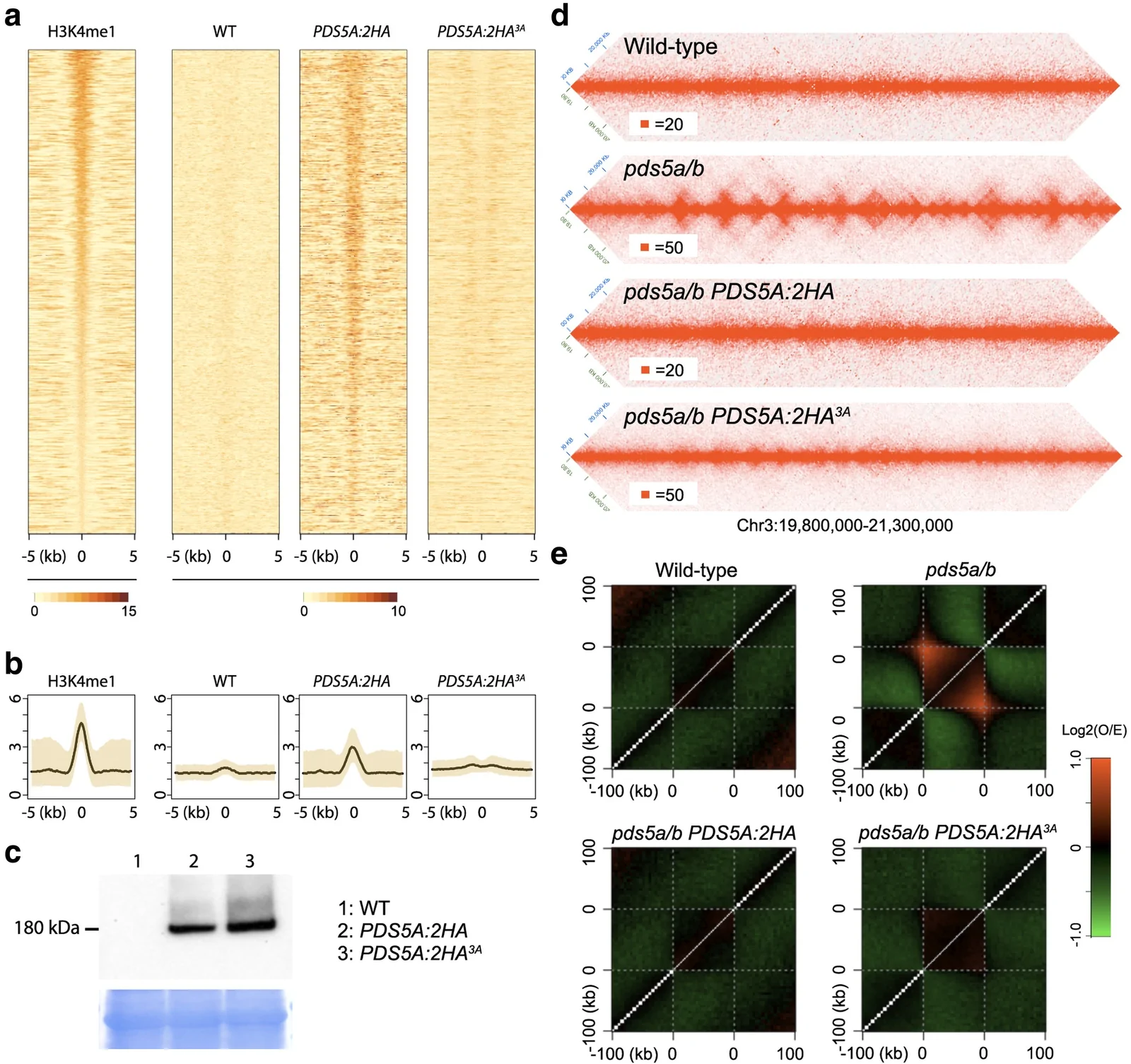
PDS5A regulates TAD-like domain formation independently of interacting with chromatin. a, b) Normalized ChIP-seq reads distribution around chromatin regions marked with H3K4me1. The heatmaps were generated from the same set of genomic regions, which were first ranked by H3K4me1 signal and then used to display different ChIP-seq datasets side by side. The curves in panel b) show median values, and the shaded areas indicate the interquartile range. The H3K4me1 plot was generated by re-analyzing anti-H3K4me1 ChIP-seq data (Niu et al. 2021). WT, *PDS5A:2HA*, and *PDS5A:2HA^3A^* indicate anti-HA ChIP-seq reads, with WT serving as a non-specific binding control. c) Protein expression of *PDS5A:2HA* and *PDS5A:2HA^3A^*. Total protein from shoots of 14-d-old WT, *PDS5A:2HA* and *PDS5A:2HA^3A^* plants was analyzed by western blot, the membrane was probed with anti-HA antibody and stained with Coomassie Blue to verify equal loading of total protein across lanes. A band corresponding to PDS5A was detected in both tagging lines at the expected size of 180 kDa. d) Comparison of Hi–C maps of different genotypes. The graphs illustrate Hi–C maps of a 1.5 Mb representative genomic region, normalized at 5 kb resolution. e) Metagene plot depicting relative chromatin contact strengths within TAD-like domains and their 100 kb flanking regions. Relative chromatin contact was calculated as the log2 ratio of observed versus expected contact frequencies (log2(O/E)), based on normalized Hi–C matrices.

### Fine-scale chromatin architectural stripes and loops emerge at promoters

On mammalian Hi–C maps, 2 prominent signatures of loop extrusion are “corner peaks” (enriched focal contacts at TAD borders or loop anchors) and architectural stripes extending inward from these peaks, reflecting cohesin-driven loop formation and CTCF-defined barriers (Rao et al. 2014; Vian et al. 2018). To investigate fine-scale chromatin contact patterns in *pds5a*, we employed the Micro-C method (Hsieh et al. 2015; Sun et al. 2024), which not only recapitulated TAD-like domains in *pds5a*, but also enabled investigating its genome-wide DNA interactions at a nucleosomal resolution (Figure S7). When analyzed at a resolution of 1 kb or higher, we found that the *pds5a* Micro-C map displayed widely distributed stripes and dots, which were barely observable in wild-type Micro-C data (Fig. 5a). In this study, we use the term “stripe” purely as a descriptive label for a specific visual enrichment pattern and do not intend to imply a conserved CTCF-based mechanism in *Arabidopsis*. These stripe and dot features, which largely overlapped (Fig. 5b), were predominantly localized at TAD-like domain boundaries, suggesting that they emerge from the dynamic interplay of cohesin-mediated loop extrusion and the presence of genomic or chromatin features that constrain loop expansion.

**Figure 5:**
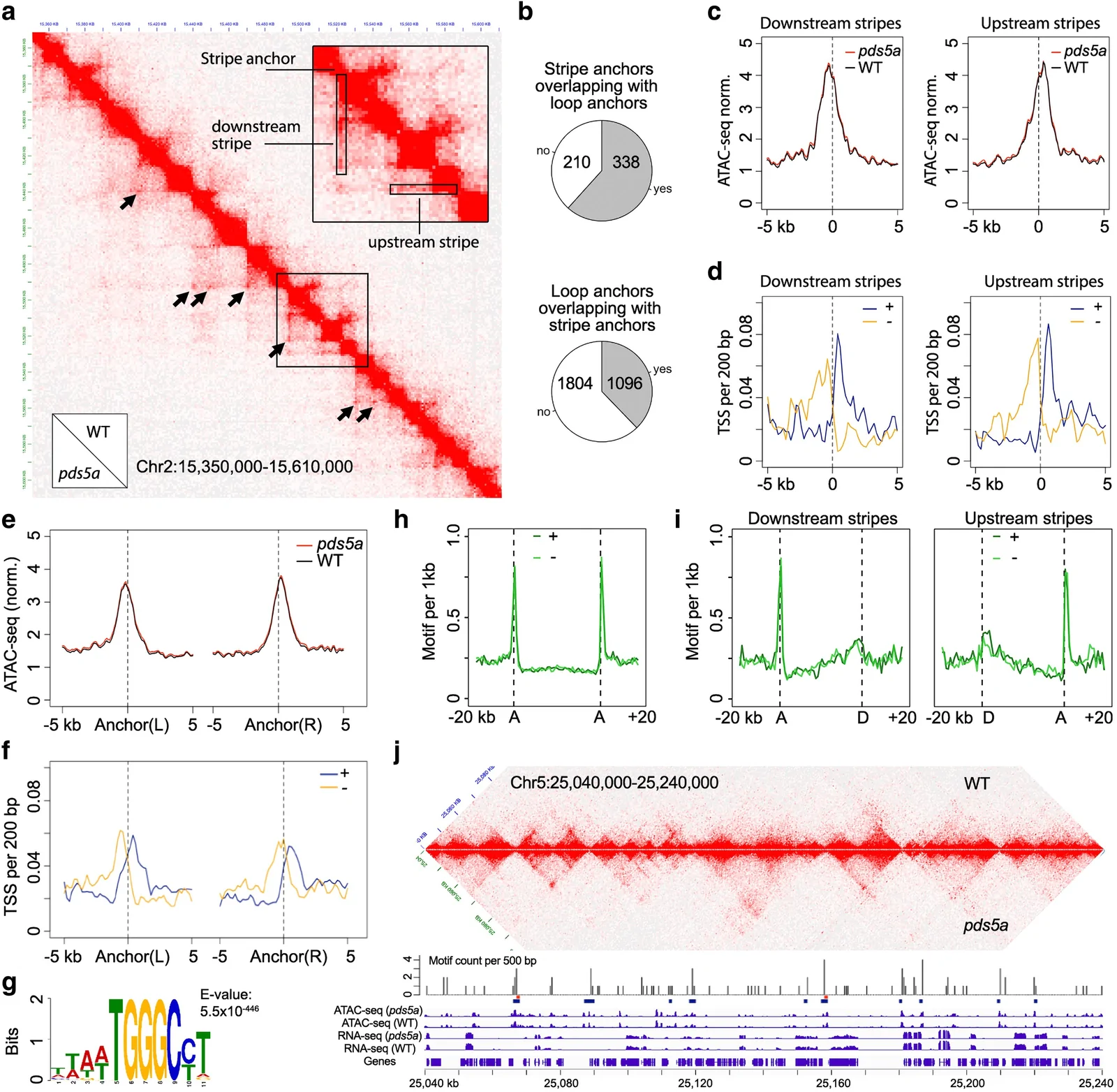
Characterization of stripe anchor and chromatin loop anchor regions. a) Example showing stripes (inset) and chromatin loops (indicated by arrows) as detected in the *pds5a* Micro-C map normalized to 1 kb resolution. b) Substantial overlap between stripe anchor regions and loop anchor regions. c) Comparison of chromatin accessibility at stripe anchors in *pds5a* mutant and wild-type samples, shown with normalized ATAC-seq read coverage. d) Density of TSSs per 200 bp at stripe anchors, shown separately for plus-strand (blue) and minus-strand (orange) transcripts. e) Chromatin accessibility at loop anchors in *pds5a* mutant and wild-type samples, presented as normalized ATAC-seq read coverage. For each chromatin loop, the interacting regions are labeled as L (left) and R (right) according to their genomic coordinates. f) Density of TSSs per 200 bp at loop anchors, separated by plus-strand (blue) and minus-strand (orange) transcripts. g) Sequence logo representing the overrepresented motif identified at loop anchor regions. h, i) Density profiles showing motif enrichment (motifs per 1 kb) around loop anchors (h) and stripe regions (i). The presence of the site-II motif (5′-TGGGCC/T-3′) is shown for the plus strand (dark green) and minus strand (light green) separately. In these plots, anchor regions and stripe region termini are labeled as “A” (anchor) and “D” (distal end), respectively. j) Integrated overview of stripes and loops within a representative 200 kb genomic region, displayed alongside tracks for various genomic features. The motif count track indicates the number of site-II motifs on both strands. Blue and red segments below the motif count track denote loop anchor and stripe anchor regions, respectively.

To systematically identify and analyze stripes, we implemented an adapted version of the Zebra algorithm, which was originally developed for mammalian Hi–C data (Vian et al. 2018) (see Methods for details; Figure S8a). Applied to 1 kb resolution Micro-C maps of *pds5a* samples, this pipeline identified 548 stripes ranging from 1 to 4 kb in length (Figure S8b). Each stripe originated from an “anchor” region and typically extended 30 to 40 kb into the adjacent “stripe domain” (Figure S8c). Integration with *Arabidopsis* epigenomic datasets revealed that stripe anchors co-localized with regions of high chromatin accessibility (Fig. 5c). Furthermore, meta-analysis showed that stripe anchors were preferentially located near outward-facing TSSs, overlapping with TAD-like domain borders, and were significantly enriched for highly expressed genes while depleting inactive genes (Fig. 5d; Figure S8d and e).

On the *pds5a* Micro-C maps, we also observed distinct focal interaction peaks (“dots”) that represent enriched pairwise contacts between specific genomic loci (Fig. 5a). In this study, we refer to these peaks as chromatin loops, and to their underlying loci as loop anchors. In total, we identified 1,677 such chromatin loops using the SIP tool (Rowley et al. 2020), which was specifically developed to detect such local interaction peaks based on their enrichment over the surrounding neighborhood, rather than relying solely on a global statistical model of genome-wide background contacts (see Methods; Data Set S2). Notably, loop anchors shared several features with stripe anchors, including overlapping within highly accessible chromatin regions and TAD-like domain borders, and enrichment at promoters of genes with high expression levels (Fig. 5e and f; Figure S8d and e).

In mammals, chromatin extrusion is halted by transcription factor CTCF at specific loci that contain CTCF motifs (de Wit et al. 2015; Guo et al. 2015; Sanborn et al. 2015). From *pds5a* Micro-C data, a particularly interesting finding was the strong enrichment of the TGGGC(C/T) motif, known as the plant “site-II” element, at stripe anchors and loop anchors (Fig. 5g–j). This was also the only motif revealed by our de novo sequence analysis. The site-II motif is enriched in *Arabidopsis* promoter regions, and it has in vivo interactions with members of several types of transcription factor families including TCP, TRB, and LBD (Giraud et al. 2010; Xu et al. 2018; Amiard et al. 2024). Although we cannot infer causality at this stage, the striking co-localization of loop and stripe anchors with the plant-specific site-II motif implies that this sequence element, along with its associated transcription factors, functions as a candidate regulatory factor that may act analogously to mammalian CTCF by demarcating boundaries or stabilizing loop anchors during cohesin-mediated loop extrusion.

### Distal promoters function as enhancers under extended chromatin looping

Chromatin loops in *pds5a* broadly strengthen intra-chromosomal contacts among highly accessible promoters, however, they are neither enriched nor depleted for DEGs (Figure S9a). Despite this, we asked whether some distal promoters brought into proximity in cis might be associated with regulatory effects. We selected 3 *pds5a*-upregulated genes whose promoters form new cis contacts with distal loci, which we refer to here as potential enhancers (Fig. 6a–c, Figure S9b-d). Mutant lines harboring T-DNA insertions adjacent to these candidate enhancers and within the loop-forming region were crossed with *pds5a* to test their impact on target gene expression.

**Figure 6:**
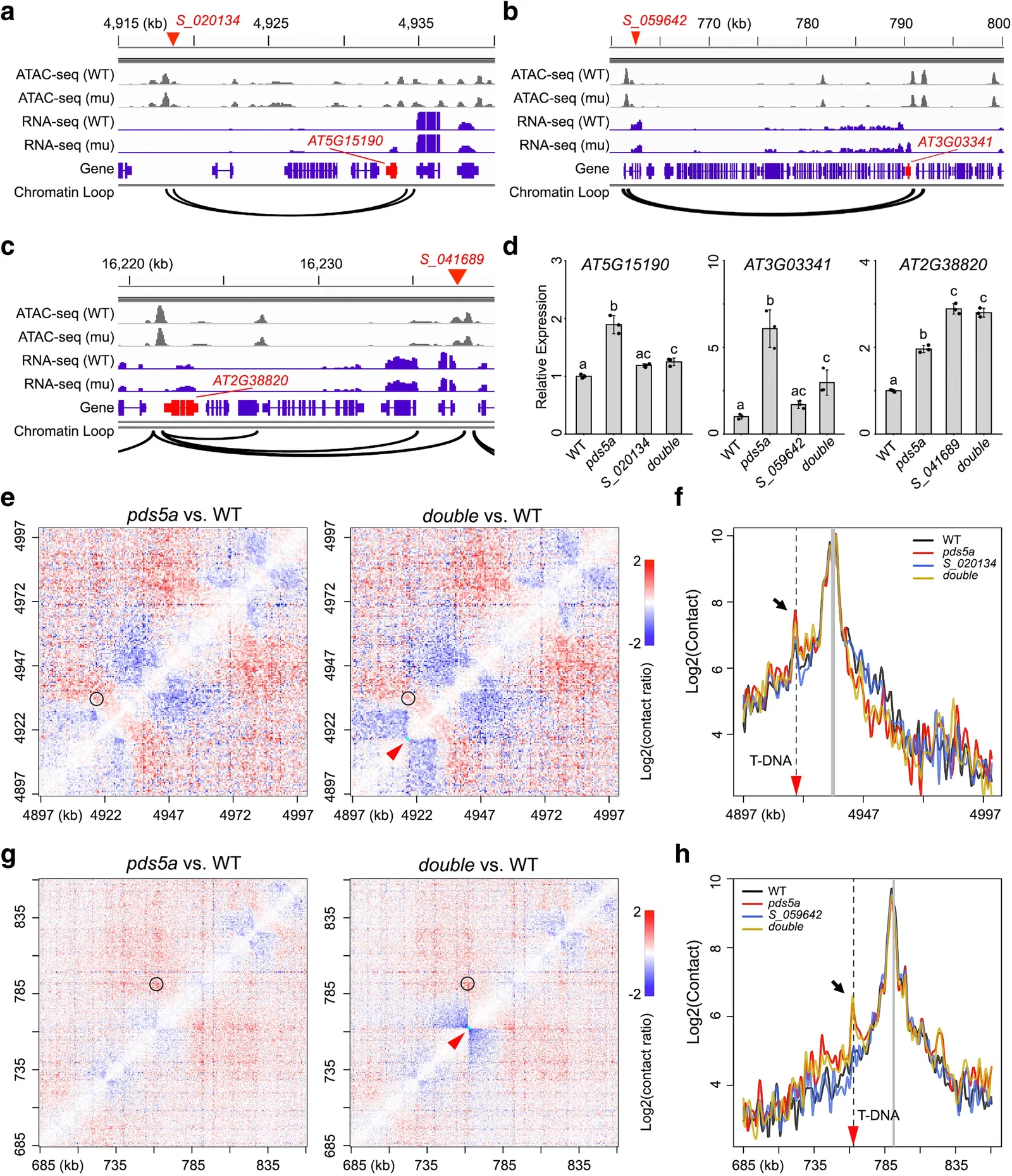
Newly established cis-chromatin contacts in *pds5a* influence gene expression. a–c) Genomic regions containing *pds5a*-dependent chromatin loops (arcs) whose anchor regions overlap with genes that are upregulated in *pds5a* (gene models in red). Gene expression and chromatin accessibility in these regions are shown by RNA-Seq and ATAC-seq tracks, respectively. WT, wild type; mu, *pds5a*. In each panel, the triangle marks the insertion site of the SALK line used in this study. d) Gene expression in various genetic backgrounds, expressed as mean ± standard deviation of 3 biological replicates. For each set of comparisons, “double” refers to the double mutant. Different letters above the bars indicate statistically significant differences at *P* < 0.05 determined by one-way analysis of variance followed by Tukey's post hoc test. e) Comparison of Micro-C contact maps at 500 bp resolution. All maps were generated from Capture Micro-C data, and the full genomic region covered by the capture probes is shown. The arrowhead points to the T-DNA insertion site; the circle highlights the chromatin loops indicated in panel (a). f) Chromatin contacts between the promoter (marked by a vertical bar) and its surrounding regions, derived from Capture Micro-C. The arrow indicates the chromatin loops described in panel (a). The dotted line, together with the arrowhead, marks the T-DNA insertion site. Genotype labels are as in panel (d). g, h) As in panels e) and f), respectively, but for the genomic region containing the chromatin loops described in panel (B).

For 2 of the 3 genes analyzed (*AT5G15190* and *AT3G03341*), the *pds5a*-dependent up-regulation was suppressed by the T-DNA insertion (Fig. 6d). For *AT2G38820*, the T-DNA insertion next to the potential enhancer itself caused strong up-regulation, which may have masked any additional *pds5a*-dependent effect; therefore, this gene was not analyzed further. For the other 2 genes, we performed Capture Micro-C to map their chromatin interaction networks in different genetic backgrounds (Figure S10). The Capture experiments achieved over 200-fold enrichment of Micro-C reads in the target genomic regions, enabling high sequencing depth across multiple samples at substantially reduced cost. As expected, since T-DNA increased the physical distance between loci that span the insertion site, a strong decrease in chromatin contacts over the T-DNA locus was observed; other than that, we did not find drastic changes in chromatin contact patterns in Capture Micro-C probed regions (Fig. 6e and g). In particular, T-DNA insertion adjacent to their potential enhancers did not disrupt the respective chromatin loops (Fig. 6e and g). Next, we examined promoter chromatin contact in detail by extracting their Micro-C contacts. Interestingly, independent of *PDS5A*, the T-DNA insertion itself did not alter promoter–enhancer contact patterns (Fig. 6f and h). It is worth noting that, in these 2 cases, the T-DNA insertions are located approximately 15 and 25 kb away from the respective target genes, which is considerably farther than typical distances (∼1 kb) reported for *Arabidopsis* enhancer-promoter pairs (Zhao et al. 2022; Tan et al. 2023). Our data, although limited to 2 loci, show that *pds5a*-dependent chromatin loops can bring distal promoters into proximity with their target genes and that local T-DNA insertion next to the distal element alters transcription without grossly perturbing the loop contact pattern. These 2 examples imply that *PDS5A* mutation may extend the functional range of cis-regulatory communication in *Arabidopsis*.

## Discussion

In this work, genetic and biochemical data establish *PDS5A* as a key negative regulator of cohesin-dependent chromatin extrusion in somatic *Arabidopsis* cells. Loss of *PDS5A* markedly strengthens local insulation and TAD-like domains and induces abundant short-range stripes and focal loops, which are further enhanced by *WAPL1/2* loss and are fully dependent on *SYN4* (Fig. 2, Fig. 7). This chromatin extrusion regulatory system resembles that in vertebrates, where *WAPL* depletion substantially extends chromatin loop length (Haarhuis et al. 2017). However, in *Arabidopsis*, *PDS5A* clearly dominates over *WAPL*: mutations in *WAPL* alone have little impact on TAD-like domains, whereas *pds5a* already suffices to reveal a robust loop-extruded layer, and *pds5a wapl1 wapl2* further extends long-range chromatin contacts (Fig. 2a). Together with the recovery of cohesin components in PDS5A:2HA IP-MS from *Arabidopsis* and the Y2H evidence for candidate PDS5A contacts with SCC3 and SMC3 (Figs. 2b and 3), these findings suggest that PDS5A acts as the major negative regulator of loop extension in *Arabidopsis*, likely by promoting cohesin release from chromatin. PDS5 proteins are highly conserved across eukaryotes, and vascular plants in particular harbor duplicated PDS5A/B-type homologs as well as more diverged PDS5C-like proteins that arose later in seed plant evolution (Quiroz et al. 2025). Plant PDS5A-type proteins retain a large Armadillo-type fold domain and a Tudor domain, whereas PDS5B and PDS5C-type homologs show greater sequence divergence and evidence of lineage-specific neofunctionalization (Quiroz et al. 2025). Such sequence divergence may underlie the specific role of PDS5A in regulating chromatin architecture, making it functionally unique among *Arabidopsis* PDS5 homologs. In the future, functional experiments probing PDS5A-chromatin and PDS5A-cohesin (particularly SYN4-containing cohesin) interactions in other plant species, together with mechanistic studies in *Arabidopsis*, will be required to reveal how broadly this regulatory module is conserved across plants and how it operates in species with prominent TAD-like domains. The function of SYN4 in regulating genome folding was reported in recent work, where *syn4* mutants show broad alterations in intra- and inter-chromosomal contacts, particularly at telomere-proximal regions (Pirita Paajanen 2024). In the present study, we show that introducing *syn4*, but not *syn2*, into *pds5a* abolishes TAD-like domains and restores chromatin contact decay to wild-type-like profiles (Fig. 2a). Thus, among mitotic kleisins (SYN2 and SYN4), SYN4-containing cohesin complexes appear to be the principal engines for loop formation that becomes visible once PDS5A-mediated unloading is relaxed.

**Figure 7:**
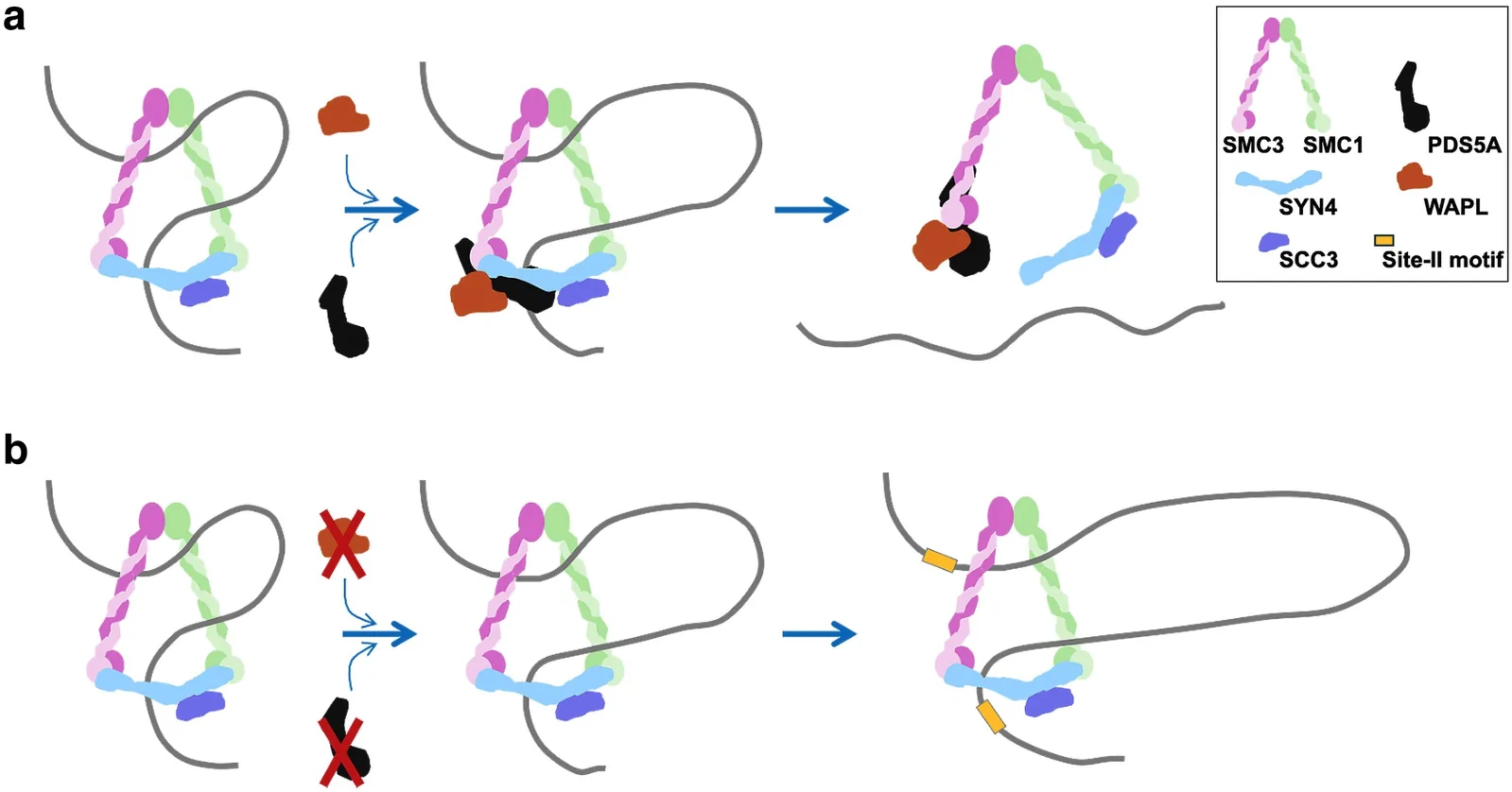
A proposed model for the influence of PDS5A on loop extrusion. a) In the presence of PDS5A, chromatin is released from the cohesin complex, and loop extrusion ceases. This balance between cohesin loading and unloading restricts the formation of long-range chromatin contacts, which become more pronounced in *pds5a*. WAPL proteins also inhibit chromatin extrusion; however, PDS5A exerts the predominant effect. b) In the absence of PDS5A, the cohesin complex is stably bound to chromatin and loop extrusion proceeds further, bringing distant loci into physical proximity and resulting in visible TAD-like structures in the *pds5a* background. The anchors of newly formed loops and architectural stripes are enriched with the site-II motif (TGGGC(C/T)).

Notably, in the *PDS5A* Tudor-mutant line, loss of H3K4me1 binding severely reduces PDS5A chromatin association yet fully restores wild-type-like contact decay and suppresses TAD-like domains, suggesting that PDS5A-mediated unloading of cohesin and repression of loop extension occur independently from PDS5A-H3K4me1 interactions (Fig. 4). Furthermore, PDS5A-chromatin interaction patterns turned out to be unrelated to TAD-like domain borders (Figure S6). These findings point to a model in which the critical function of PDS5A in controlling chromatin loop formation lies in how it modulates SYN4-containing cohesin complexes, rather than in its chromatin-binding activity per se (Fig. 7). Future structural and mutational analyses will be needed to resolve the SYN4-containing cohesin structure and the PDS5A-cohesin interaction interface to refine this model. Concerning PDS5A-H3K4me1 interactions, it is noteworthy that *pds5a/b* double mutants exhibit increased sensitivity to DNA damage induced by γ-irradiation, and the histone mark H3K4me1 has been reported to recruit DNA repair factors in plants (Pradillo et al. 2015; Quiroz et al. 2024). Together, these observations suggest that PDS5A-H3K4me1 interactions may contribute to DNA damage repair, potentially by facilitating the recruitment or stabilization of repair machinery at chromatin.

Comparing the transcriptome of different *pds5a* tissues revealed that DEGs do not preferentially reside inside, outside, or at the borders of TAD-like domains, and insulation scores at regions harboring DEGs remain largely unchanged relative to the genome background (Fig. 1; Figure S3). This is consistent with studies showing that perturbation of higher-order chromatin architecture has limited transcriptome consequences (Nora et al. 2017; Rao et al. 2017; Schwarzer et al. 2017). It should be noted that our gene expression analyses were performed at the tissue level. Recent work showed that cohesin loss alters coexpression patterns of cis-linked genes in single cells, whereas these effects are less apparent at the cell-population level (Dong et al. 2024). Thus, the potential impact of *pds5a* TAD-like domains or chromatin loops on gene expression in individual nuclei remains unclear. However, for the 2 chromatin loops we present in this study, which have T-DNA insertions near loop anchor regions, the T-DNA abrogates *pds5a*-dependent transcriptional activation without disrupting local loop structures (Fig. 6f and h). These insertions alter gene expression only in the context of *pds5a*-dependent chromatin loops, suggesting that extrusion-driven juxtaposition in *Arabidopsis* may facilitate regulatory interactions between distal promoters and target genes. However, we cannot establish or exclude a causal relationship between chromatin looping and gene expression based on these T-DNA lines. Instead, CRISPR-based approaches that mutagenize chromatin anchor regions will be required to directly test whether chromatin loop formation can be disrupted and whether it is sufficient to alter transcription. Overall, we propose that the primary role of extrusion-mediated domains in plants is not to globally alter transcription but to modulate the spectrum of specific long-range regulatory elements. In wild-type, strong PDS5A activity keeps extrusion short-ranged, constraining most cis-regulatory communication within local promoter neighborhoods, whereas in *pds5a*, extended loops expose genes to additional distal promoters that can either activate or repress transcription depending on their regulatory content.

In conclusion, our findings place PDS5A and SYN4-containing cohesin as key components of a plant-specific chromatin extrusion module that generates TAD-like structures and cis-regulatory loops when cohesin unloading is relaxed. Our work helps reconcile the apparent discrepancies in TAD patterns among different plant species (Domb et al. 2022) and aligns plant 3D genome organization with conserved loop extrusion principles observed across eukaryotes. Future efforts to manipulate PDS5A and SYN4 in other plant species and developmental contexts should clarify the extent to which extrusion-driven loops are exploited for adaptive gene regulation, and whether tuning cohesin unloading can be used to engineer chromatin topology and transcriptional outputs in plants. In parallel, a key priority will be to functionally characterize the site-II motif and its binding factors, among which TCP, TRB, and LBD proteins currently appear to be strong candidates that co-localize with loop and stripe anchors. Systematic identification and perturbation of this motif and the corresponding binding factors will be essential to test whether they act as plant-specific chromatin boundary or anchor factors analogous to CTCF. Furthermore, biochemical reconstitution and in vivo tethering experiments could determine whether these factors directly modulate cohesin residence time or processivity, thereby providing a mechanistic link between sequence-specific DNA binding and extrusion-driven 3D genome architecture in plants.

## Materials and methods

### Plant materials and growth conditions

The wild-type reference used in this study is the *A. thaliana* accession Columbia (Col-0). The T-DNA insertion mutant lines *pds5a-1* (*SALK_114556*), *wapl1-1* (*SALK_076791*), *wapl2-1* (*SALK_127445*), *syn2* (*SALK_044851*), *syn4* (*SALK_076116*), *pds5b-1* (*SALK_092843*), *SALK_020134*, *SALK_059642*, *SALK_041689* were obtained from the Nottingham Arabidopsis Stock Centre.

After surface sterilization, Arabidopsis seeds were sown on vertical half-strength Murashige and Skoog (1/2 MS) media plates (1% sucrose; 0.3% Phytagel). Following a 72 h stratification at 4 °C, the plants were grown in growth chambers (MLR-352-PE from PHCbi) at 21 °C in long-day conditions (16 h light/8 h dark). Germinating seeds were collected 24 h after they were placed under long-day conditions. Heat stress treatment was performed by transferring 11-d-old plants to a growth chamber set at 37 °C by day; the other parameters remained identical. Heat-stressed plants were harvested 3 d later for subsequent analyses.

### Plasmid construction

The coding sequences (CDSs) of the selected cohesin complex proteins (SCC3, SYN4, SMC1, SMC1_N & C termini, SMC3, SMC3_N & C termini) were amplified by PCR using HiQu polymerase and cloned into GAL4 activation domain (pGADT7) vectors. Similarly, the coding sequence of the PDS5A C-terminus was amplified and cloned into the GAL4 DNA-binding domain (pGBKT7) vector by Gibson Assembly. Recombinant plasmids were transformed into *Escherichia coli*, and positive clones were verified by plasmid sequencing. The oligonucleotide primers used for cloning are provided in Data Set S6.

The construct for *PDS5A:2HA*^3A^ was based on *PDS5A:2HA*, which we reported previously (Göbel et al. 2024). Mutagenesis PCR was performed using oligos listed in Data Set S6 to introduce mutations at 3 amino acids in the Tudor domain of PDS5A (W1377A, Y1384A, Y1403A).

### In situ Hi–C

*In situ* Hi–C libraries were prepared essentially following a previously described protocol (Liu et al. 2017). Two independent Hi–C libraries were generated per sample, each starting from approximately 0.3 g of crosslinked material, homogenized for nuclei extraction. The isolated nuclei were resuspended in 150 µl 0.5% SDS and divided into 3 aliquots. After incubation at 62 °C for 5 min, 145 µl water and 25 µl 10% Triton X-100 were added to quench SDS, followed by incubation at 37 °C for 15 min. Chromatin digestion was carried out overnight at 37 °C with 50 U *Dpn*II (NEB) per tube. The next day, *Dpn*II was inactivated at 62 °C for 20 min. Subsequently, sticky ends were filled in using 1 µl each of 10 mM dTTP, dATP, dGTP, 10 µl 1 mM biotin-14-dCTP (Jena Bioscience), 29 µl water, and 40 U Klenow fragment (Thermo Fisher Scientific) at 37 °C for 2 h. Proximity ligation was performed by adding 663 µl water, 120 µl 10× blunt-end ligation buffer (300 mM Tris-HCl, 100 mM MgCl_2_, 100 mM DTT, 1 mM ATP, pH 7.8), and 40 U T4 DNA ligase (Thermo Fisher Scientific) at room temperature for 3 h. The resulting nuclei were collected by centrifugation, resuspended in 650 µl SDS buffer (50 mM Tris-HCl, 1% SDS, 10 mM EDTA, pH 8.0), and treated with 10 µg proteinase K (Thermo Fisher Scientific) at 55 °C for 30 min, followed by reverse crosslinking in 30 µl 5 M NaCl at 65 °C overnight.

DNA was isolated, treated with RNase A (Thermo Fisher Scientific) at 37 °C for 30 min, and purified. Hi–C DNA (1 µg) was adjusted to 130 µL in TE buffer (10 mM Tris-HCl, 1 mM EDTA, pH 8.0) and sheared to fragments with an average size of 500 bp using a Q800R3 sonicator (QSONICA; 25% amplitude, 15 s on/off pulses, total 4.5 min). Fragments larger than 300 bp were recovered with Ampure beads. For biotin removal from unligated ends, DNA in a 50 µl reaction was incubated with 0.5 µl of 10 mM dTTP, 0.5 µL 10 mM dATP, and 5 U T4 DNA polymerase (Thermo Fisher Scientific) at 20 °C for 30 min, followed by another Ampure cleanup. End repair and adaptor ligation were performed with the NEBNext^®^ Ultra™ II DNA Library Prep Kit (NEB). Streptavidin C1 beads (Invitrogen) were used for affinity purification, and the libraries were PCR-amplified prior to sequencing with 2 × 150 bp reads on an Illumina NovaSeq 6000 platform.

### Micro-C

Plant tissues were fixed, and nuclei were isolated as described (He et al. 2024). Following the second crosslinking step with disuccinimidyl glutarate (DSG), nuclei were washed in MBuffer (10 mM Tris-HCl, pH 8.0, 50 mM NaCl, 5 mM MgCl_2_, 1 mM CaCl_2_, 0.1% Triton X-100, and 1× EDTA-free Complete protease inhibitor cocktail; Roche). Micrococcal nuclease (MNase) titration assays and Micro-C-XL library preparation were then performed essentially as described (Metelova et al. 2023) with the following adaptations. MNase (NEB) digestion was performed in MBuffer at 37 °C for 10 min (500 rpm), and optimal conditions yielding predominantly mono- and di-nucleosomal fragments were determined by agarose gel electrophoresis. Digestion was stopped with 4 mM EGTA and incubation at 65 °C for 10 min. Nuclei were washed in 1× NEBuffer 2.1 containing 0.1% Triton X-100 and resuspended in Micro-C master mix 1 (10 µL 10× NEBuffer 2.1, 2 µL 100 mM ATP, 5 µL 100 mM DTT, 67 µL H_2_O, 5 µL T4 PNK at 10 U/µL, 1 µL 500 mM EGTA) and incubated at 37 °C for 15 min (500 rpm). Subsequently, 10 µL of 5 U/µL Klenow fragment was added and incubated for 15 min at 37 °C. Biotin labeling was performed using Micro-C master mix 2 (10 µL 1 mM biotin-dATP, 10 µL 1 mM biotin-dCTP, 1 µL 10 mM dTTP/dGTP mix, 5 µL 10× T4 DNA ligase buffer, 0.25 µL 200× BSA, 23.75 µL H_2_O) for 45 min at 25 °C. Reactions were stopped with 9 µL 0.5 M EDTA and incubated at 65 °C for 20 min. Proximity ligation was carried out in Micro-C master mix 3 (50 µL 10× T4 DNA ligase buffer, 425 µL H_2_O, 25 µL T4 DNA ligase) for 2.5 h at room temperature with rotation. Nuclei were then treated with Micro-C master mix 4 (10 µL 10× NEBuffer 1.1, 85 µL H_2_O, 5 µL Exonuclease III) at 37 °C for 15 min. Chromatin was reverse crosslinked overnight at 65 °C using Proteinase K (12.5 µL, 20 mg/mL), 6.25 µL 20% SDS, and 5 µL 5 M NaCl. DNA was purified by phenol–chloroform extraction, RNase-treated, and further cleaned using the ChIP DNA Clean & Concentrator Kit (Zymo Research). Di-nucleosomal DNA was then size-selected using the PippinHT system (Sage Science) according to the manufacturer's instructions. Finally, sequencing libraries were prepared from the size-selected DNA using the NEBNext Ultra II DNA Library Prep Kit (NEB).

### Capture micro-C

BAC plasmids (F8M21, T21P5, and T17B22) served to generate probes for capturing Micro-C, which were labeled with biotin-16-dUTP (Roche) via nick translation. Labeled probes were pooled into a 20 µL hybridization mix (Twist Bioscience) for a final concentration of 0.5 ng/µL for each BAC. Separately, 300 ng of each indexed Micro-C library (NEBNext Ultra II DNA Library Prep Kit) was mixed with 0.6 µL NEXTFLEX Universal blockers (Revvity) in 12 µL library solution. The NEXTFLEX universal blockers are short DNA oligonucleotides that hybridize to adapter sequences at the ends of library molecules. They were used during target enrichment to prevent concatemerization of library fragments. The hybridization and streptavidin pull-down steps followed the Twist Target Enrichment Hybridization v2 Protocol (Twist Bioscience): after denaturation at 95 °C and cooling, probe and library solutions were combined and topped with Hybridization Enhancer solution. Hybridization was performed at 70 °C for 16 h; products were captured with Dynabeads MyOne Streptavidin C1 (Invitrogen), washed at 68 °C and 48 °C, eluted in Tris-HCl (pH 8.0), and PCR-amplified for 14 cycles with P5 and P7 primers.

### Hi–C and micro-C data processing

Read mapping to the TAIR10 reference was performed using Bowtie 2 (v2.2.4) (Langmead and Salzberg 2012), followed by PCR duplicate removal and filtering. Hi–C reads were filtered according to our previously described procedures, which took into account the distribution of the restriction enzyme recognition site (5′-GATC-3′) to ensure that only *bona fide* Hi–C ligation products were retained. In contrast, all inter-chromosomal Micro-C reads were retained, as were all intra-chromosomal reads for which the mapped distance between read1 and read2 exceeded 500 bp in the reference genome. Hi–C and Micro-C reads filtering statistics for all samples are provided in Data Set S1. Iterative matrix correction for map normalization was performed with the HiTC R package (Servant et al. 2012); matrix normalization was stopped when the *eps* value fell below 1 × 10^−4^. Additionally, Hi-C files for interactive visualization were generated using Juicer, on the filtered, informative Hi–C and Micro-C reads (Durand et al. 2016).

### Chromatin insulation, TAD-like domains and chromatin loop annotation

Insulation scores were calculated according to a previous study (Göbel et al. 2024). We used Hi–C maps normalized with 5 kb bins, and intrachromosomal contacts up to 100 kb to compute insulation scores. Potential “insulated regions” were identified by searching for regions with local insulation score minima, which was done by using the peak function from the splus2R package in R with span = 10. In this study, we annotated the regions with insulation scores lower than −0.25 and overlapping with insulation score local minima as “insulated regions”. A list containing insulation scores and insulated region annotations is available in Data Set S2.

TAD-like domains were annotated using the Hi–C contact map from *pds5a* leaf tissues, normalized at 5 kb resolution. The arrowhead algorithm, which we previously applied to similar chromatin features in various plant species, was used for domain identification (Liu et al. 2017; Karaaslan et al. 2020; Göbel et al. 2024). For this study, the TAD score matrix cutoff was set to 0.95, candidate TADs were required to contain at least 6 filtered pixels, and the minimum TAD score at domain borders was set to 1.10. Differential TAD-like domains relative to the *pds5a* leaf sample were identified using the hicDifferentialTAD function from the HiCExplorer tool (Ramírez et al. 2018). Because not all *pds5a* TADs were found in the genome in a side-by-side manner, for differential TAD calling, only *P*-values corresponding to “in-TAD” chromatin contacts were retained. *P*-values were corrected for multiple testing using the Benjamini–Hochberg (BH) procedure, and TAD-like domains with BH-adjusted *P* < 0.01 were classified as differential. The complete differential TAD-like domain annotation is available in Data Set S2.

Chromatin loops were called using SIP (v1.6.5.) (Rowley et al. 2020) with the following parameters: “*-norm KR -g 1.5 -min 2.0 -max 2.0 -mat 5000 -d 6 -res 1000 -sat 0.01 -t 2800 -nbZero 6 -factor 1 -fdr 0.01 -del true -cpu 1 -isDroso false*” on Micro-C maps of *pds5a* normalized at 1 kb resolution.

For all types of annotation, the following pericentromeric regions were excluded: Chr1 (11,500,001 to 17,700,000), Chr2 (1,100,001 to 7,200,000), Chr3 (10,300,001 to 17,300,000), Chr4 (1,500,001 to 6,300,000), and Chr5 (9,000,001 to 16,000,000).

### Stripe annotation

Stripes were detected using the Zebra algorithm (Vian et al. 2018). For each chromosome, both ICE-normalized and raw contact matrices were used. Contacts between loci separated by more than 400 kb were excluded. For ICE-normalized matrices, all zero values were also set to NA. For each pixel (*i, j*), in each matrix of size *n* × *n*, the following metrics were computed:

1. Sum of raw counts and median of ICE-normalized values in a local window: To quantify local interaction strength around the given pixel (*i, j*), the sum of raw contact counts (Sum_Local_) within a window of fixed size was calculated:
  $$\begin{array}{rl} indices_{local(horizontal)} & =\{i-w÷2,i-w÷2+1,\ldots ,i+w÷2\}\\ indices_{local(vertical)} & =\{\;j-w÷2,j-w÷2+1,\ldots ,j+w÷2\} \end{array}$$

where *w* is the window size and ÷ denotes integer division. The sum of values in the row or column over these indices represents the horizontal or vertical local interaction signal, respectively. If the pixel (*i, j*) had fewer than 5 reads, the local sum was set to NA.

Additionally, for the ICE-normalized matrix, the median value across the same local window is computed as Median_local_. For stripe detection in 1 kb matrices, the window size w was set to 5 pixels.

2. Median of ICE-normalized values of neighborhood: To estimate a background signal away from the pixel (*i, j*), the median ICE-normalized values were calculated in 4 neighborhood regions: top, bottom, left, and right (Median_direction_). Each region was defined using a fixed window *f* and a gap *g* between the pixel (*i, j*) and the neighborhood, defined as:
  $$\begin{array}{c} indices_{left}=\{\;j-f-g,j-f-g+1,\ldots ,j-g-1\}\\ indices_{right}=\{\;j+g+1,j+g+2,\ldots ,j+f+g\}\\ indices_{top}=\{i-f-g,i-f-g+1,\ldots ,i-g-1\}\\ indices_{bottom}=\{i+g+1,i+g+2,\ldots ,i+f+g\} \end{array}$$

where *j* and *i* denote column and row indices, respectively. For stripe detection in 1 kb matrices, the window size *f* was set to 4 pixels and the gap *g* to 2 pixels.

3. Bias and expected value: Next, the horizontal and vertical biases for both horizontal and vertical local regions were defined as:
  $$Bias=Sum_{local}/Median_{local}$$

which were used to compute expected values for individual directions (top, bottom, left, and right) as:


$$Expected_{direction}=Median_{direction}/Bias$$

with top and bottom using the horizontal bias and left and right using the vertical bias.

4. Statistical testing: Poisson tests were performed for each pixel to evaluate whether the observed local contact count is significantly greater than the expected value derived from its neighborhood. The tests were applied as follows: For testing for downstream stripes, the observed value was the horizontal *Sum_local_*, and 2 tests were performed against *Expected_top_* and *Expected_bottom_*. For upstream stripes, the observed value was the vertical *Sum_local_*, and 2 tests were performed against *Expected_left_* and *Expected_right_*. A pixel was considered potentially part of a stripe if both tests in the corresponding directions satisfied the following thresholds: FC (Fold change) > 1.1 and *P*-value < 0.005. Pixels meeting these conditions were assigned a value of 1 in a binary matrix of size *n* × *n*. All other pixels were assigned 0.

Next, stripe regions were identified from the binary enrichment matrix by scanning along each row (for downstream stripes) and each column (for upstream stripes) to find consecutive runs of pixels with value 1. Run-length encoding (RLE) was applied to each row or column to identify these runs. Runs were selected as candidate stripes if their length met or exceeded 5 pixels, and their row (or column) index and start/end positions were recorded. Adjacent candidate stripes on the same row or column were merged if separated by a gap less than or equal to 3 pixels, with merging performed by extending the end position of the first stripe. After merging, only stripes with a total length above 11 pixels were retained. For vertical stripes, those located in adjacent columns, and for horizontal stripes, those in adjacent rows, were grouped if their positions differed by 2 bins or fewer. Each group was then consolidated by recording the minimum and maximum values for row/column, start, and end positions. Finally, stripes anchored within the following pericentromeric regions were excluded: Chr1 (11,500,001 to 17,700,000), Chr2 (1,100,001 to 7,200,000), Chr3 (10,300,001 to 17,300,000), Chr4 (1,500,001 to 6,300,000), and Chr5 (9,000,001 to 16,000,000).

### *De novo* motif analysis

DNA motifs within the *pds5a* chromatin loop were identified by first extracting sequences from accessible chromatin regions (ATAC-seq peaks) located within the loop anchors. For this, the 2 peak sets from *pds5a* replicates 1 and 2 were merged based on overlapping peaks with the “findOverlaps” function from the R package GenomicRanges (Lawrence et al. 2013). Peaks within stripe anchors were extracted and only peaks fully contained within an anchor region were retained for downstream analysis. Sequences corresponding to these filtered peaks were then extracted from the TAIR10 reference genome FASTA file.

The sequences were submitted to MEME for de novo motif discovery (Bailey et al. 2015) with the following settings: “*-dna -oc. -nostatus -time 14400 -mod anr -nmotifs 3 -minw 4 -maxw 12 -objfun classic -revcomp -markov_order 0*”.

### Chromatin immunoprecipitation and data analyses

Arabidopsis shoot tissue was fixed under vacuum for 25 min at room temperature in 2 mM DSG in PBS (50 mM potassium phosphate; 150 mM NaCl; pH 7.2) buffer supplemented with 0.01% Triton X-100. Subsequently, formaldehyde stock solution was added to achieve a 1% final concentration, and the samples were further incubated under vacuum for 25 min. To quench the fixation, the solution was replaced with 0.15 M glycine in PBS buffer, and samples were incubated under vacuum for an additional 10 min at room temperature. Approximately 0.5 g of the fixed tissue was then homogenized and resuspended in nuclei isolation buffer (20 mM HEPES, pH 8.0; 250 mM sucrose; 1 mM MgCl_2_; 5 mM KCl; 40% glycerol; 0.25% Triton X-100; 0.1 mM PMSF; 0.1% 2-mercaptoethanol). The isolated nuclei were pelleted and subsequently resuspended in 0.5 mL sonication buffer (10 mM potassium phosphate, pH 7.0; 0.1 mM NaCl; 0.5% sarkosyl; 10 mM EDTA). Chromatin was fragmented by sonication using a QSONICA Q800R3 sonicator, resulting in an average fragment size of approximately 500 bp. Fifty microlitres of 10% Triton X-100 were added to the sonicated chromatin, and the mixture was further mixed with an equal volume of IP buffer (50 mM HEPES, pH 7.5; 150 mM NaCl; 5 mM MgCl_2_; 10 µM ZnSO_4_; 1% Triton X-100; 0.05% SDS) and incubated with Pierce™ Anti-HA magnetic beads (Thermo Fisher Scientific) for 2 h at 4 °C. Following immunoprecipitation, the beads were washed at 4 °C as follows: twice with IP buffer, once with IP buffer containing 500 mM NaCl, and once with LiCl wash buffer (0.25 M LiCl; 1% NP-40; 1% sodium deoxycholate; 1 mM EDTA; 10 mM Tris-HCl, pH 8.0), each for 3 min. Beads were then briefly washed with TE buffer (10 mM Tris-HCl, pH 8.0; 1 mM EDTA). For elution, the beads were resuspended in 200 µL elution buffer (50 mM Tris-HCl, pH 8.0; 200 mM NaCl; 1% SDS; 10 mM EDTA) and incubated at 65 °C for 6 h, followed by treatment with Proteinase K (Thermo Fisher Scientific) at 45 °C for 1 h. DNA was subsequently purified using the MinElute PCR Purification Kit (QIAGEN) and was either analyzed by qPCR or used to generate sequencing libraries with the NEBNext^®^ Ultra™ II DNA Library Prep Kit (NEB). The libraries were sequenced with 2 × 150 bp reads on an Illumina NovaSeq 6000 platform.

ChIP-seq reads were mapped to the TAIR10 genome using Bowtie 2 (Langmead and Salzberg 2012). H3K4me1 ChIP-seq analysis was conducted by processing the raw sequencing data described in a previous study (Niu et al. 2021), and peak calling was done with MACS2 v.2.1.1 (Zhang et al. 2008) using parameters “*-g 1.2e8 --bw 450 --keep-dup=1*”. The enriched genomic regions in different plant materials are tabulated in Data Set S5.

### Gene expression analyses

RNA sequencing (RNA-Seq) was performed with 2 replicates per sample. Total RNA was extracted from unfixed seedlings, roots, and germinating seeds by mechanically disrupting the plant material in liquid nitrogen, followed by RNA extraction using the RNeasy Plant Mini Kit (Qiagen), according to the manufacturer's instructions. For each sample, 500 ng of total RNA was treated to remove residual genomic DNA by adding 0.5 μL 10× reaction buffer (100 mM Tris, pH 7.5, 25 mM MgCl_2_, 1 mM CaCl_2_), 0.5 μL DNase I (Thermo Scientific), and 0.5 μL RNaseOUT (Thermo Scientific) and incubating at 37 °C for 30 min. The reaction was stopped by adding 0.5 μL 50 mM EDTA and incubating at 65 °C for 10 min. RNA-seq libraries were generated from the DNase-treated RNA using the NEBNext^®^ Ultra™ RNA Library Prep Kit for Illumina (NEB), NEBNext^®^ Poly (A) mRNA Magnetic Isolation (NEB), and NEBNext Multiplex Oligos for Illumina, following the manufacturer's protocol at 0.5× reaction volume. The libraries were sequenced with 2 × 150 bp reads on an Illumina NovaSeq 6000 platform.

Sequencing reads were mapped to the TAIR10 reference genome using TopHat2 (v2.1.1) (Kim et al. 2013) with the “--no-novel-juncs” option. A count table with the number of reads per gene was generated by overlapping the aligned reads with TAIR10 gene annotations using the R package GenomicAlignments (Lawrence et al. 2013). Differential gene expression analysis was performed using the R package DESeq2 (Love et al. 2014). DEGs were identified based on a false discovery rate smaller than 0.01 and a log2 fold change of greater than 1.5. DEGs are listed in Data Set S3.

For analysis of individual gene expression, total RNA was extracted from the samples using the RNeasy Plant Mini Kit (Qiagen) according to the manufacturer's protocol. The RNA was subsequently treated with DNase I (Thermo Scientific) to eliminate residual genomic DNA. Afterwards, first-strand cDNA was synthesized from the RNA using the qScriber™ cDNA Synthesis Kit (highQu). Quantitative RT-PCR was performed on an Applied Biosystems™ QuantStudio™ qPCR System (Thermo Fisher Scientific) employing qPCRBIO SyGreen Mix ROX (Lo-ROX) (PCR Biosystems) and gene-specific primers listed in Data Set S6.

### Chromatin accessibility analyses

For ATAC-seq, approximately 0.1 g of fixed plant material was chopped on ice in 1 mL general purpose buffer (GPB; 0.5 mM spermine, 30 mM sodium citrate, 20 mM MOPS, 80 mM KCl, 20 mM NaCl, 0.5% (v/v) Triton X-100, pH 7.0) and filtered first through single-layer Miracloth (Merck Millipore, with pore size 22 to 25 μm) and then through a cell strainer. DAPI was added to a final concentration of 600 nM, and 50,000 diploid nuclei were sorted into NIB by fluorescence-activated cell sorting (FACS) based on DAPI signal, using a Bio-Rad S3e cell sorter.

ATAC-seq was performed with 2 biological replicates. Nuclei were first incubated at 60 °C for 5 min and centrifuged at 1,000*×g* at 4 °C for 5 min. To the nuclei pellet, 10 μl TD, 1 μl TDE1 (Nextera^®^ DNA Library Prep kit), and 9 μL water were added, and the reaction was incubated at 37 °C for 30 min. The reaction was then adjusted to 200 μL with SDS buffer (50 mM Tris, pH 8.0, 1% (w/v) SDS, 10 mM EDTA), and 8 μl 5 M NaCl was added. After incubating overnight at 65 °C, DNA was purified using the Qiagen Mini-purification kit (Qiagen). To prepare DNA for sequencing, 2 μL of DNAwase used as a template for PCR using the NEBNext^®^ High-Fidelity 2× PCR Master Mix (NEB) and Nextera index primers (Illumina). The following PCR protocol was applied: 72 °C for 5 min, followed by initial denaturation at 98 °C for 20 s, 12 cycles of denaturation at 98 °C for 10 s, annealing at 63 °C for 30 s, and extension at 72 °C for 1 min, finally 72 °C for 1 min. DNA was then size-selected using magnetic beads (SpeedBead Magnetic Carboxylate Modified Particles, Cytiva) to recover fragments between 200 and 500 bp.

ATAC-seq reads mapping was performed according to our previous study (Hu et al. 2019). BigWig and count files were generated using deepTools (v3.5.5) with “bamCoverage” and BEDTools (v2.31.1) with the “multicov” function, respectively. Accessible peaks were defined from ATAC-seq data using MACS2 (v2.1.1-python-2.7.12) peak calling with a genome size of 1.2 × 10^8^ and duplicate reads limited to 1 (--keep-dup=1).

### Protein extraction and western blot

Total protein was extracted from 14-d-old aerial tissue using TRIzol-chloroform and subsequently dissolved in a buffer containing 88 mM Tris-HCl (pH 6.8), 4.4 M urea, 2.2% SDS, 10 mM DTT, and 11% glycerol. The protein samples were analyzed by western blot, and HA-tagged proteins were detected by anti-HA-HRP (1:2,500; sc-7392, Santa Cruz Biotechnology). The chemiluminescent signals were detected with HRP substrate (Takara) in the iBright CL750 Imaging System (Thermo Fisher Scientific).

### Isolation of PDS5A-interacting proteins

Shoot tissue of 14-d-old plants was fixed for 30 min under vacuum with 1% formaldehyde in MC buffer (10 mM potassium phosphate, pH 7.0, 50 mM NaCl, 0.1 M sucrose). The fixative was replaced with 0.15 M glycine in MC buffer, wherein the plant material was incubated under vacuum for an additional 10 min. Approximately 0.5 g fixed tissue was homogenized in nuclei isolation buffer (20 mM Hepes, pH 8.0, 250 mM sucrose, 1 mM MgCl2, 5 mM KCl, 40% glycerol, 0.25% Triton X-100, 0.1 mM PMSF, 0.1% 2-mercaptoethanol). The homogenate was filtered through double-layered miracloth (Millipore). The isolated nuclei were washed in NIB and subsequently resuspended in 0.5 mL sonication buffer (10 mM potassium phosphate, pH 7.0, 0.1 mM NaCl, 0.5% sarkosyl, 10 mM EDTA). The samples were sonicated 5 times for 10 s, with a resting period of 50 s between each sonication with the sonicator (QSONICA Q800R3) set at 40% of full capacity. After the addition of 50 µL 10% Triton X-100 the sonicated samples were mixed with an equal volume of IP-buffer (50 mM HEPES, pH 7.5; 150 mM NaCl; 5 mM MgCl_2_; 10 µM ZnSO_4_; 1% Triton X-100; 0.05% SDS) and Pierce™ Anti-HA magnetic beads (Thermo Fisher Scientific). The IP was carried out at 4 °C for 30 min and the beads subsequently collected and washed as described in chromatin immunoprecipitation. Finally, the beads were resuspended in TE buffer, mixed with 5× Laemmli Loading Buffer, and boiled at 95 °C for 10 min. The proteins were separated on a 10% SDS-PAGE gel and submitted to the Core Facility Hohenheim for proteomic analysis.

### Mass spectrometry

Nano-LC-ESI-MS/MS experiments were performed on an Ultimate 3000 RSLCnano system (Dionex, Thermo Fisher Scientific) coupled to an Orbitrap Exploris 480 mass spectrometer (Thermo Fisher Scientific) using a Nanospray Flex source (Thermo Fisher Scientific). Tryptic peptides were directly injected to a precolumn (µ-precolumn C18 PepMap100, 300 µm, 100 Å, 5 µm × 5 mm, Thermo Fisher Scientific) and an analytical column (NanoEase M/Z HSS C18 T3, 1.8 µm 100 Å 75 µm × 250 mm column, Waters GmbH) operated at 35 °C. Gradient elution was performed at a flow rate of 300 nl/min using a 30 min gradient with the following profile: 2% - 55% solvent B in 30 min, 55% - 95% solvent B in 10 min, 5 min isocratic at 95% solvent B, 10 min from 95% to 2% B and then 10 min reequilibration with 2% B. Solvents used were 0.1% formic acid (solvent A) and 0.1% formic acid in 80% acetonitrile (80/20, v/v) (solvent B). The Orbitrap Exploris 480 and the Ultimate 3000 were operated under the control of XCalibur software (version 4.7.69.37) and Sii Xcalibur (version 1.8.0.530) (Thermo Fisher Scientific). Internal calibration was performed using lock-mass ions from ambient air as described in (Olsen et al. 2005). Survey spectra (*m/z* = 200 to 2,000) were detected in the Orbitrap at a resolution of 60,000 at *m/z* = 200. Data-dependent MS/MS mass spectra were generated for the 30 most abundant peptide precursors in the Orbitrap using high-energy collision dissociation (HCD) fragmentation at a resolution of 15,000 with normalized collision energy of 30.

For data analysis, Mascot 2.6 (Matrix Science) was used as a search engine for protein identification. Spectra were searched using the *A. thaliana* protein database retrieved from UniProt (www.uniprot.org as of September 2024). Database searching was conducted using a no-enzyme setting, with peptide precursor mass tolerance set to 5 ppm and fragment ion tolerance set to 0.02 Da. Methionine oxidation and Phosphorylation of serine, threonine, and tyrosine were allowed as variable modifications, and Carbamidomethylation of cysteine residues was set as a fixed modification.

### Yeast two-hybrid assays

Sequence-confirmed BD and AD constructs were co-transformed into the Y2HGold yeast strain. Transformants were first selected on DDO (SD/-Leu/-Trp) medium to confirm the presence of both bait and prey plasmids. Positive colonies were subsequently subjected to a drop assay on DDO, TDO (SD/-His/-Leu/-Trp) supplemented with 1 mM 3-amino-1,2,4-triazole (3-AT), and QDO (SD/-Ade/-His/-Leu/-Trp) media. Yeast growth on selective media was used to evaluate protein–protein interactions, with growth on TDO and QDO indicating activation of reporter genes and a positive interaction between PDS5A and the cohesin proteins.

## Supplementary Material

koag325_Supplementary_Data

## Data Availability

Short read data of in situ Hi–C, Micro-C, ChIP-seq, ATAC-seq, and RNA-seq are publicly available at NCBI Sequence Read Archive under accession number PRJNA1392550. Large datasets, such as normalized Hi–C and Micro-C matrices, BigWig ChIP-seq and ATAC-seq track files, are available in the figshare repository. They are accessible with Digital Object Identifier (DOI) 10.6084/m9.figshare.30814679.

## Code availability

All scripts used in this study are available upon request.
