# Supplementary material for: PDS5A restricts SYN4-dependent long-range chromatin contacts to shape TAD-like chromatin architecture in Arabidopsis
Source: Plant Cell. 2026 Oct 27;38(11):koag325. doi: 10.1093/plcell/koag325 (PMC13739617; doi:10.1093/plcell/koag325)
Supplement: Supplementary file 1 [file koag325_supplementary_data.zip › supp_figs_v3.docx]

**PDS5A restricts SYN4-dependent long-range chromatin contacts to shape TAD-like chromatin architecture in *Arabidopsis***

Sofia Tzourtzou, Zhidan Wang, Sarah Leuchtenberg, Priyanka Kumari, David Latrasse, Moussa Benhamed, Chang Liu

**Supplementary Figure 1.** Comparison of *Arabidopsis* chromatin domains. **A)** Size distribution of TAD-like domains in *pds5a* (identified from 5kb resolution Hi-C map) and compartment domains (CDs) identified from wild-type 1 kb resolution Hi-C map. p-values indicate the statistical significance as determined by two-sided Wilcoxon rank-sum tests. **B)** Venn diagram showing genomic regions (Mb) covered by each type of domain annotation. **C)** Hi-C maps of a 2 Mb genomic region of wild-type and *pds5a* at 5 kb resolution. Annotated domains in this region are shown as segments. The CD annotation is according to (Shu et al. 2024).

 **Supplementary Figure 2.** Chromatin organization features exhibit distinct responses under heat stress. The Hi-C contact maps, normalized to a 20 kb bin size, illustrate a 3 Mb segment of chromosome 3. Arrows highlight long-range chromatin interactions occurring between two KNOT ENGAGED ELEMENT regions, previously characterized in an earlier study (Grob et al. 2014).

**Supplementary Figure 3.** Gene expression and insulation scores change at TAD boundaries in different *pds5a* tissues and conditions. **A)** Distribution of differential TAD-like domains (diff_TADs) and differentially expressed genes (DEGs) in *pds5a*. For each sample type, the left Venn diagram shows the genomic regions (Mb) covered by TAD-like domains, and the right Venn diagram shows the numbers of DEGs located outside (Out) or inside TAD-like domains. **B)** Genomic distribution of differentially expressed genes in relation to TAD-like domains in *pds5a*. "Border," "Inside," and "Outside" indicate genes located at TAD-like domain borders, within TAD-like domains, and in the regions between TAD-like domains, respectively, which were defined in the *pds5a* leaf sample. "All genes" serves as a background reference and includes all genes in the genome. **C)** Boxplots show the distribution of insulation scores at TAD boundaries across tissues or conditions in wild-type (grey) and *pds5a* (green), with the box spanning the interquartile range (IQR; Q1-Q3), the horizontal line indicating the median, whiskers extending to 1.5×IQR, and outliers shown as individual points. P-values were calculated using paired Wilcoxon signed-rank tests and corrected using the Holm method within each genotype. **D)** Boxplots of insulation scores (top panels) and log2(RPKM) expression values (bottom panels) at TAD boundaries overlapping transcription start sites (TSS) of DEGs (|log2 fold-change| > 1.5 and p-value <= 0.01) in *pds5a*. Comparisons are shown for *pds5a* heat, root, and germinating seed samples versus mock. TAD boundaries were selected based on TSS overlap within a 2 kb bin. Sample sizes (n) indicate the number of selected TAD boundaries or genes per comparison. Statistical testing used paired, two-sided Wilcoxon signed-rank tests with Holm correction for insulation scores and gene expression values separately. **E)** Genome-wide correlation between change in insulation score and change in gene expression at all TSS. Scatterplots show Pearson’s correlation coefficient (r) between log2 fold-change of gene expression and the corresponding change in insulation score at each TSS.

**Supplementary Figure 4.** TAD-like domains in *pds5a* are independent of endoreduplication cycles. **A)** Endopolyploidy measurements in wild-type (WT) and *pds5a* seedlings. Nuclei were isolated from 14-day-old seedlings and the ploidy distribution was analyzed with the S3e Cell Sorter (Bio-Rad). **B)** TAD-like structures in *pds5a* are present in both nuclei with and without completing endoreduplication cycles. The Hi-C maps (normalized at a 5 kb bin size) show a 0.5 Mb genomic region from chromosome 3.

**Supplementary Figure 5.** Effects of WAPL depletion on chromatin organization in mammalian cells. **A)** Comparison of Hi-C maps of a 10 Mb representative genomic region from mouse embryonic stem cells before (WAPL 0 h) and 96 hours after WAPL depletion (WAPL 96 h). The Hi-C maps were reconstructed from a public dataset (Liu et al. 2025) and normalized at bin size of 20 kb. **B)** Genome-wide intrachromosomal-contact frequency plotted as a function of genomic distance.

**Supplementary Figure 6.** Characterization of PDS5A-chromatin interactions. **A)** Venn diagram showing the overlap of genomic regions (Mb) enriched in individual PDS5A:2HA ChIP-seq replicates. **B)** Venn diagram comparing genomic regions (Mb) enriched for PDS5A:2HA (red) and H3K4me1 (black). H3K4me1-enriched regions were identified from (Niu et al. 2021). **C)** PDS5A-chromatin interactions positively correlate with H3K4me1 enrichment. Each dot represents a PDS5A-interacting chromatin region whose midpoint overlaps an H3K4me1 peak. Spearman’s correlation coefficient and the corresponding p-value are indicated. **D)** Venn diagram showing the overlap of genomic regions (Mb) enriched for PDS5A:2HA (red) and PDS5A:2HA^3A^ (blue). **E)** Comparison of fold enrichment for genomic regions bound by PDS5A:2HA (red) and PDS5A:2HA^3A^ (blue). The p-value was calculated using the Mann–Whitney U test. **F and G)** Normalized ChIP-seq reads heatmap of PDS5A:2HA and the mutagenized PDS5A:2HA^3A^ at individual (F) and averaged (G) TAD-like borders (n= 1208).

**Supplementary Figure 7.** Comparison of chromatin interaction patterns presented on Hi-C and Micro-C maps. **A)** At 5 kb resolution, both Hi-C and Micro-C maps reveal the emergence of prominent TAD-like domain structures in *pds5a*. The maps show a representative 2 Mb region on chromosome 1. **B)** Micro-C maps of a 1 Mb subregion shown in (A) are displayed at 1 kb resolution. Micro-C data from a recent study are included for comparison (Sun et al. 2024). Note that the lower halves of the two comparisons come from the same dataset.

**Supplementary Figure 8.** Detection of architectural stripes in *pds5a* with the Zebra algorithm. **A)** Computational approach for stripe detection, illustrated for an upstream stripe. (1) For each pixel located at a maximum distance from the diagonal, a vertical local region is defined, including the focal pixel and its two adjacent pixels (green), and the raw contact values are summed. (2) Additionally, two flanking neighbourhood regions (Left and Right; dashed boxes), located two pixels away from the local region, are extracted. Median contact values are computed for the local and neighbourhood regions using ICE-normalised Hi-C data. (3) The expected background signal is calculated, and Poisson statistics are applied to test whether the local signal read count exceeds the expected background value. (4) Each pixel yields two fold-change (FC) values and two associated p-values (Left and Right). Pixels satisfying both FC > 1.1 and p < 0.005 in both comparisons are retained and clustered to obtain stripes. (5) Stripes are annotated as linear regions extending from the anchor point to the most distal retained pixel. For downstream stripes, the same analysis is performed using a horizontal local region and top and bottom neighbourhood zones. **B)** Distribution of stripe anchor widths. **C)** Distribution of stripe domain lengths measured from the most distant contact from the anchor. **D)** Most stripe and loop anchor regions overlap with *pds5a* TAD‑like domain borders. **E)** Enrichment of gene expression categories in stripe anchors and chromatin loop anchors relative to genomic background. Genes outside pericentromeric regions were binned by average RPKM values from *pds5a* leaf samples into six categories: non-expressed (RPKM = 0; n = 5,403), very low (0-0.5; n = 3,182), low (0.5-5; n = 6,718), moderate (5-20; n = 6,504), high (20-100; n = 3,394), and very high (>100; n = 875). Colours indicate log2(observed/expected) enrichment, with asterisks denoting statistical significance from bin-wise chi-squared goodness-of-fit tests with Benjamini-Hochberg correction (**** p < 0.0001, ** p < 0.01, * p < 0.05, ns not significant).

**Supplementary Figure 9.** Association between gene expression change and long-range chromatin contacts in *pds5a*. **A)** Overlap between differentially expressed genes in *pds5a* and loop anchor regions. A gene was defined as overlapping a loop anchor if the anchor intersected the gene body or its 1 kb flanking regions. p-values indicate results from two-tailed chi-square tests. **B to D)** Micro-C maps normalized to a 500 bp window size reveal the presence of prominent chromatin loops formed in *pds5a* (Arrows). RNA-seq coverage tracks above each map display gene expression in the corresponding genomic regions, with boxes indicating differentially expressed genes. For each panel, a magnified view of the genomic region encompassing the chromatin loop, along with gene annotations, is shown on the right; the region surrounding the T-DNA insertion is further enlarged. Chromatin loops are represented by black arcs, and differentially expressed genes are highlighted by red rectangular frames. The red triangle marks the insertion site of the SALK line used in this study. The T-DNA insertion site was confirmed by Sanger sequencing of a T-DNA-specific PCR product.

**Supplementary Figure 10.** Enrichment of Capture Micro-C libraries. **A and B)** Percentages of on-target Micro-C contacts in individual libraries, defined as reads overlapping with genomic regions covered by probes. ctr, a *pds5a* Micro-C library without hybridization-mediated capture, representing the baseline of Micro‑C contacts concerning the region of interest. **C)** Distribution of Micro-C reads in probe-captured regions and their neighboring regions. In each plot, the solid triangle denotes the BAC-targeted genomic region, while the dashed triangle indicates an adjacent region of equal size. The number of Micro-C reads corresponding to chromatin contacts within each highlighted region is shown. These plots are derived from replicate #1 of the wild-type sample and are representative of all Capture Micro-C datasets. **D)** Number of Micro-C reads in individual Capture Micro-C samples. Micro-C reads were considered as “on target” if either read1 or read2 was mapped to the genomic region covered by probes.

**References**

**Grob S, Schmid MW, and Grossniklaus U**. Hi-C analysis in Arabidopsis identifies the KNOT, a structure with similarities to the flamenco locus of Drosophila. Mol Cell. 2014:**55**(5):678–93. https://doi.org/10.1016/j.molcel.2014.07.009

**Liu NQ, Magnitov M, Schijns M, van Schaik T, Teunissen H, van Steensel B, and de Wit E**. Extrusion fountains are restricted by WAPL-dependent cohesin release and CTCF barriers. Nucleic Acids Res. 2025:**53**(12). https://doi.org/10.1093/nar/gkaf549

**Niu Q, Song Z, Tang K, Chen L, Wang L, Ban T, Guo Z, Kim C, Zhang H, Duan CG, et al.** A histone H3K4me1-specific binding protein is required for siRNA accumulation and DNA methylation at a subset of loci targeted by RNA-directed DNA methylation. Nat Commun. 2021:**12**(1):3367. https://doi.org/10.1038/s41467-021-23637-4

**Shu J, Sun L, Wang D, Yin X, Yang M, Yang Z, Gao Z, He Y, Calonje M, Lai J, et al.** EMF1 functions as a 3D chromatin modulator in Arabidopsis. Mol Cell. 2024:**84**(24):4729-4739 e6. https://doi.org/10.1016/j.molcel.2024.10.031

**Sun L, Zhou J, Xu X, Liu Y, Ma N, Liu Y, Nie W, Zou L, Deng XW, and He H**. Mapping nucleosome-resolution chromatin organization and enhancer-promoter loops in plants using Micro-C-XL. Nat Commun. 2024:**15**(1):35. https://doi.org/10.1038/s41467-023-44347-z
